# Identification of novel molecular subtypes and construction of a prognostic signature via multi-omics analysis and machine learning in lung adenocarcinoma

**DOI:** 10.3389/fonc.2025.1590216

**Published:** 2025-07-21

**Authors:** Ke Ma, Jie Xu, Congyue Wang, Xu Cao, Wenjie Yu, Jingjing Xi, Xuan Zhang, Jiamin Zhan, Yang Liu, Aoyang Yu, Shuhan Liu, Yanhua Liu, Chong Chen, Xiaoli Mai

**Affiliations:** ^1^ Department of Radiology, Nanjing Drum Tower Hospital Clinical College of Xuzhou Medical University, Nanjing, Jiangsu, China; ^2^ Institute of Hematology, Xuzhou Medical University, Xuzhou, Jiangsu, China; ^3^ Department of Oncology, The Second Affiliated Hospital of Shandong First Medical University, Taian, Shandong, China; ^4^ Department of Hematology, General Hospital of Xuzhou Mining Group, The Second Affiliated Hospital of Xuzhou Medical University, Xuzhou, Jiangsu, China; ^5^ Department of Oncology, The Affiliated Hospital of Xuzhou Medical University, Xuzhou, Jiangsu, China; ^6^ Department of Radiology, Nanjing Drum Tower Hospital, Affiliated Hospital of Medical School, Nanjing University, Nanjing, Jiangsu, China; ^7^ Department of Oncology, Xuzhou Central Hospital Affiliated to Xuzhou Medical University, Xuzhou, Jiangsu, China; ^8^ Department of Hematology, The Affiliated Hospital of Xuzhou Medical University, Xuzhou, Jiangsu, China

**Keywords:** single-cell RNA sequencing, lung adenocarcinoma, multi-omics, prognostic signature, machine learning

## Abstract

**Introduction:**

The development of high-throughput sequencing technologies and targeted therapeutic strategies has significantly improved the prognosis of lung adenocarcinoma (LUAD) patients with sensitive gene mutations. However, patients harboring rare or no actionable mutations were rarely benefit from these targeted therapies. This study aimed to identify novel molecular subtypes and construct a prognostic signature to enhance the stratification of LUAD prognosis.

**Materials and methods:**

Novel molecular subtypes of LUAD patients were identified by applying 10 distinct clustering algorithms on multi-omics data. Single-cell RNA-sequencing (scRNA-seq) data were integrated to characterize subtype-specific immune microenvironments. A multi-omics and machine learning-driven prognostic signature (MO-MLPS) was constructed in The Cancer Genome Atlas (TCGA) LUAD dataset using ten machine learning algorithms and subsequently validated across six independent datasets from the Gene Expression Omnibus (GEO) database. The robustness of the model was assessed using the concordance index (C-index), Kaplan-Meier survival analyses, receiver operating characteristic (ROC) curves, and both univariate and multivariate Cox regression analyses. We further confirmed the effects of ANLN knockdown and the expression of a domain-negative anillin protein (dnANLN) via western blotting, cell proliferation assays, flow cytometry, and transwell migration assays *in vitro*.

**Results:**

Our analysis revealed that the novel molecular subtypes exhibited differences in prognoses, biological functions, and immune infiltration profiles in LUAD. The MO-MLPS was successfully established and validated across TCGA-LUAD cohorts, six independent GEO datasets, and their composite meta-cohort. Higher risk scores from the MO-MLPS correlated with poorer prognosis in LUAD, with AUC values exceeding 0.5 at 1, 3, and 5 years across various cohorts. The signature outperformed 49 previously published prognostic signatures. Furthermore, patients classified as high risk exhibited significantly worse overall and progression-free survival than those classified as low risk. Notably, ANLN knockdown and dnANLN expression significantly inhibited cell proliferation and migration *in vitro* and enhanced the efficacy of docetaxel.

**Conclusion:**

A comprehensive analysis of multi-omics data redefines the molecular subtype of LUAD patients. The MO-MLPS derived from subtype characteristics has the potential to serve as a clinically valuable prognostic tool. Furthermore, ANLN emerges as a promising novel therapeutic target in the treatment of LUAD.

## Introduction

Lung cancer remains the leading cause of cancer-related morbidity and mortality globally (1–3). Among its subtypes, adenocarcinoma represents the predominant form of non-small cell lung cancer (NSCLC), comprising approximately 40% of all lung cancer cases (4–6). Recent advancements in molecular detection technologies and the development of targeted therapies have significantly improved overall survival for LUAD patients with sensitive mutations (7, 8). Nevertheless, only a small fraction of LUAD patients benefit from these therapies, particularly those who lack actionable driver mutations. Consequently, it is urgent to define novel LUAD molecular subgroups to facilitate the accurate prediction of disease progression and optimize targeted therapeutic strategies.

The ongoing advancements in omics technologies enable the elucidation of the molecular characteristics of various diseases at genetic, epigenetic, and transcriptomic levels (9–11), shedding light on the molecular heterogeneity of these diseases and facilitating the development of effective treatment strategies. Multi-omics analysis, which integrates multiple datasets, can provide profound insights into the molecular mechanisms underlying complex diseases as well as highlight critical associations among various omics data types (12). Unfortunately, the majority of existing molecular subtypes of LUAD are based on one single type of omics data, with limited prognostic indicators derived from multiple omics analyses. Therefore, an integrated multi-omics approach may reveal novel insights into mechanisms affecting LUAD patients with poor prognosis and identify potential therapeutic targets.

In this study, we integrated bulk RNA sequencing profiles (including mRNA, long non-coding RNA, and microRNA), genomic mutations, as well as epigenomic DNA methylation and RNA editing data to develop consensus molecular subtypes of LUAD patients using ten different multi-omics integration algorithms. We further explored subtype-specific immune microenvironment discrepancies based on single-cell sequencing data. Subsequently, we identified a total of 123 stable prognosis-related genes that were upregulated in differential subtypes, utilizing ten machine learning algorithms to construct the MO-MLPS. Our results demonstrated the robust performance of the MO-MLPS in predicting overall survival across both training and validation cohorts, establishing a strong correlation between high the MO-MLPS risk scores and poorer outcomes in LUAD patients. Moreover, we investigated the potential role of ANLN as a therapeutic target, noting that dnANLN may address the current limitations in available targeted therapies for anillin. Our study provides a foundation for refining the novel molecular subtypes of LUAD and offers an effective tool for predicting patient survival outcomes in this malignancy.

## Materials and methods

### Integrating multi-omics datasets of LUAD

Multi-omics data of LUAD were obtained from the TCGA-LUAD cohort, encompassing profiles of whole transcriptome sequencing, DNA methylation, somatic mutations, and pertinent clinical information. The expression matrix (in transcripts per kilobase million format) for mRNA, lncRNA and somatic mutations was obtained from the “TCGAbiolinks” package (13). Annotations for TCGA’s microRNA IDs were generated using the “miRBaseVersions.db” package (14). RNA editing profiles were obtained from the Synapse data repository. Patients with an overall survival duration of less than one month were excluded from analysis. Prior to comprehensive analysis, the omics data from the six dimensions were matched each other via sample IDs. Multi-omics data integration was performed according to established protocols (15). Briefly, continuous variable gene features were filtered utilizing the “getElites” function from the “MOVICS” package, with the “method” parameter set to “mad” to select the top 1,500 genes exhibiting the greatest variability. For the analysis of binary gene mutation data, the “oncoPrint” function from the “maftools” package was initially employed to identify the top 5,000 genes with the highest mutation levels. Subsequently, the “getElites” function was utilized with the “method” parameter adjusted to “freq” to isolate the top 5% of genes with the highest mutation frequency. By integrating clinical data, genes that demonstrated statistical significance (*p* < 0.05) were identified as prognostic markers. These six dimensions were included for further analysis in the study.

### Multi-omics consensus ensemble analysis

To determine the optimal number of subtypes for LUAD patients, the “get ClustNum” function from the “MOVIC” package was utilized to estimate the number of clusters (15). With the integration of clustering prediction indexes (CPI), gaps statistics, and silhouette score, LUAD patients were ultimately classified into two distinct subtypes. The clustering process was conducted through ten clustering algorithms using the “getMOIC” function, including Cancer Integration via Multikernel Learning (CIMLR), Consensus Clustering, Similarity Network Fusion (SNF), iClusterBayes, Perturbation Clustering for data Integration and disease Subtyping (PINSPlus), moCluster, NEMO, Integrative Non-negative Matrix factorization (IntNMF), Contrastive Captioners (COCA), and Low-Rank Approximation (LRA), following the methodologies established by Niu et al. (16). The integration of clustering results from the ten algorithms, accomplished through the “getConsensusMOIC” function, improved the robustness of the consensus subtypes, leading to the final clustering outcome. In the process, the “distance” parameter of “getConsensusMOIC” was configured to “euclidean”, while the “linkage” parameter was set to “average”.

### Survival analysis

Survival curves were fitted using the Kaplan-Meier formula in the “survival” package, and visualizations were generated using the “ggsurvplot” function from the “survminer” package.

### Gene expression data of GSE cohorts preprocessing

Six independent datasets and their clinic information were retrieved from the GEO database (http://www.ncbi.nlm.nih.gov/geo) as external validation cohort, including GSE30219 (17), GSE31210 (18), GSE37745 (19), GSE42127 (20), GSE50081 (21) and GSE72094 (22). All array data underwent preprocessing through the robust multiarray averaging (RMA) algorithm and were annotated using the “SeqMap” package (23). Patients with an overall survival less than 30 days were excluded. Validation datasets were merged, with batch effects corrected, normalization performed, and log2 transformation completed through the “limma” and “sva” packages.

### Differential gene expression and functional enrichment analysis

Differentially expressed genes (DEGs) were identified using the “limma” package among the different novel subtypes. Gene set enrichment analyses (GSEA), Gene Ontology (GO), and Kyoto Encyclopedia of Genes and Genomes (KEGG) analyses were performed to explore the biological functions of DEGs via the “clusterProfiler” package (24).

### Collection, quality control and annotation of scRNA-seq data

Single-cell RNA sequencing data from 12 LUAD samples were acquired from the GSE171145 cohort in GEO database and the PRJCA001731 cohort from the China National Center for Bioinformation. Base on the consistency of consensus subtypes, seven LUAD samples were classified into subtype 1, while five samples were classified into subtype 2. Data processing and visualization were performed using the “Seurat” package. Three quality control criteria were applied to the raw data matrix: genes expressed in at least 200 and at most 10,000 single cells, cells expressing between 100 and 80,000 genes, and single cells containing fewer than 20% mitochondrial genes. All mitochondrial and ribosomal genes were excluded to enhance insight into protein-coding genes. The UMI count data were normalized to 10,000 per cell and then log-transformed. Then, Principal Component Analysis (PCA) was performed based on the top 5,000 hypervariable genes. To correct batch effects among samples, the “RunHarmony” function from the “harmony” R package was performed using default parameters before clustering analysis. Uniform manifold approximation and projection (UMAP), t-distributed stochastic neighbor embedding (t-SNE) algorithms, and cell clustering were executed using the top 20 PCs. Cell annotation was carried out through a mixed automated approach using “SingleR”, with manually corrections based on known marker genes (25).

### Cell-to-cell communication analysis

The “Cell Chat” package, a tool for analyzing intercellular communication, was used in our study to identify major signaling pathways for each novel LUAD subtypes, along with their outgoing, incoming, and overall communication patterns (26).

### Establishment and assessment of a consensus multiple machine learning algorithms-driven prognostic signature

Ten machine learning algorithms, including CoxBoost, stepwise Cox, Least Absolute Shrinkage and Selection Operator (Lasso), Ridge, Elastic Net (Enet), survival support vector machines (survival-SVM), supervised principal components (SuperPC), generalized boosted regression models (GBM), partial least Cox (plsRcox), and Random Forest (RSF), were utilized for constructing the MO-MLPS. Methodological details were derived from previously published methodology (27). Specifically, 100 genes that were upregulated for each subtype were identified as candidate genes. Subsequently, univariate Cox analysis of candidate genes was performed to screen significant prognosis-related genes in TCGA-LUAD cohort, which were then used to further construct the prognostic signature. With TCGA-LUAD as the training set and six GSE datasets as validation sets, 100 combinations were utilized to construct the predictive prognostic model, selecting the signature with the highest C-index as the MO-MLPS. Risk levels were calculated for patients across different cohorts based on the MO-MLPS and categorized into high and low-risk groups. The prognostic significance of the signature was evaluated through Kaplan-Meier curves and time-dependent C-index curves via “survminer” and “survival ROC”. Moreover, 49 LUAD-associated prognostic signatures have already published were retrieved and calculated the risk score for each patient. The prognosis predictive ability of all signatures was assessed by the C-index in different cohort.

### Analyses of tumor microenvironment infiltration

TME cell infiltration levels were calculated via the “IOBR” package. The ssGSEA algorithm was employed to calculate scores for 28 immune cell subtypes, reflecting TME infiltration and inflammatory status. Six immune subtypes were identified according to the expression profile of all solid tumors in TCGA.

### Statistical analysis

Standard Student’s t-tests were employed for pairwise comparisons, while one-way ANOVA was utilized for multiple group comparisons. A significance threshold of *p* < 0.05 was set for all statistical methods. Data analysis and figure generation were conducted using R v4.3.1, RStudio, and GraphPad Prism v10.0 software. Notations include ns for *p* > 0.05; * for *p* < 0.05; ** for *p* < 0.01; *** for *p* < 0.001.

### Experimental reagents

Details regarding experimental reagents are listed in **Supplementary Table 11**. Further methodological details associated with *in vitro* experiments are available in the **Supplementary Methods**.

## Result

### Identification of multi-omics-based consensus survival prognosis-related molecular subtypes of LUAD

**Figure 1:**
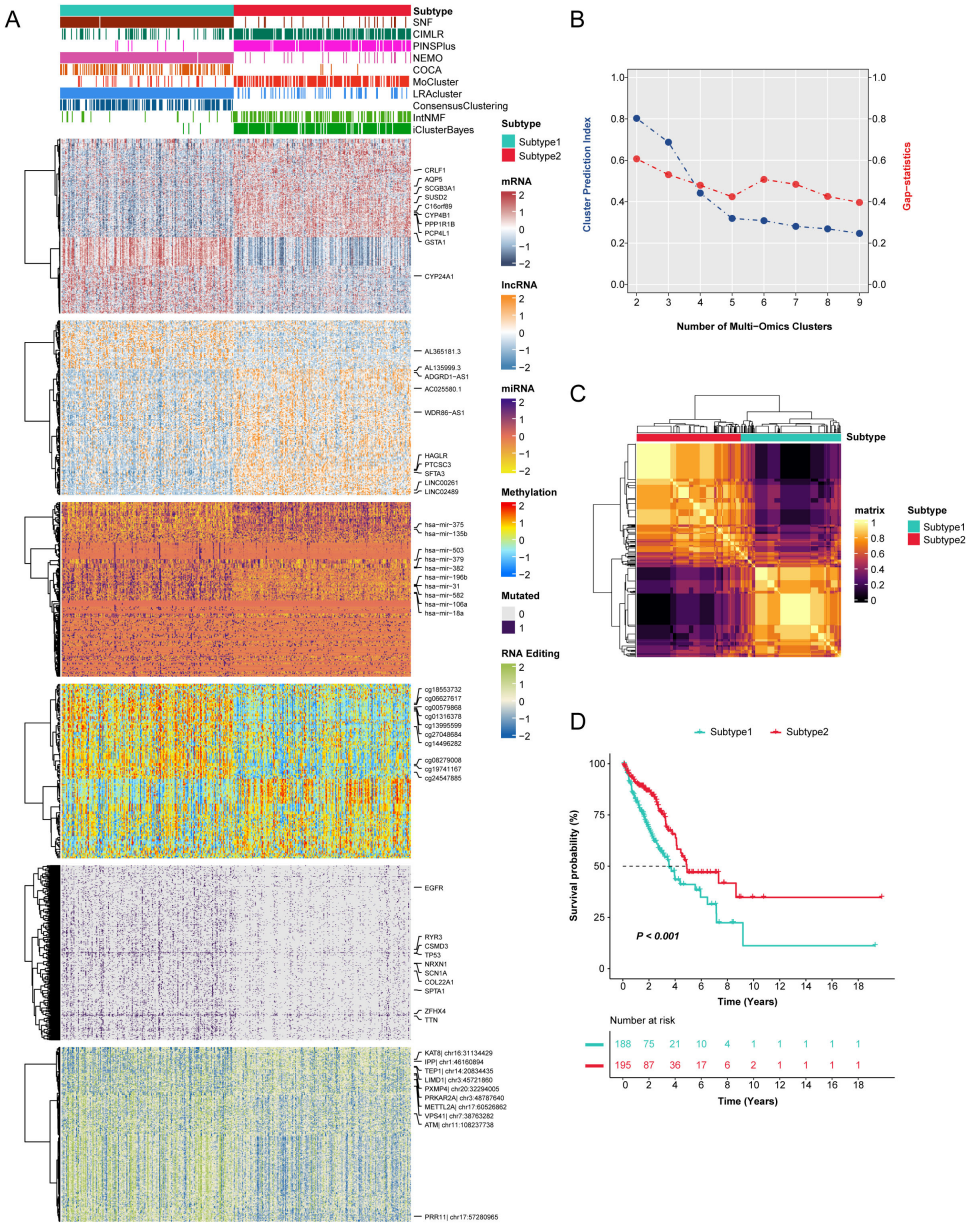
The novel integrative consensus subtypes of LUAD identified through multi-omics analysis. **(A)** Comprehensive heatmap of novel integrative subtypes clustered through 10 cutting-edge multi-omics clustering algorithms in LUAD patients, including mRNA, lncRNA, miRNA, DNA methylation site, mutant gene and RNA editing event. **(B)** The cluster prediction index and gap statistical analysis of the multi-omics subtypes. **(C)** Consensus clustering matrix for two novel prognostic subtypes based on the 10 clustering methods. **(D)** Survival difference was observed among the two novel subtypes.

When identifying novel disease subtypes, the selection of clustering methods often varies depending on individual researcher preferences, focusing primarily on individual-omics data (16, 28). To address this limitation, we employed ten ensemble clustering algorithms to independently characterize prognostic subtypes of LUAD. Our comprehensive analyses led to the identification of two novel subtypes, substantiated through the integration of Cluster prediction index, Gap statistics, and Silhouette score. The clustering results were further integrated through consensus ensemble approach with different molecular expression profiles across transcriptomic, epigenetic methylation, somatic mutations, and RNA editing events (**Figures 1A-C**). Our classification demonstrated a significant relation to overall survival (OS) (**Figure 1D**), revealing that subtype 1 was associated with poorer prognoses compared to subtype 2.

### Partitioning and characterization of integrative consensus molecular subtypes in LUAD

**Figure 2:**
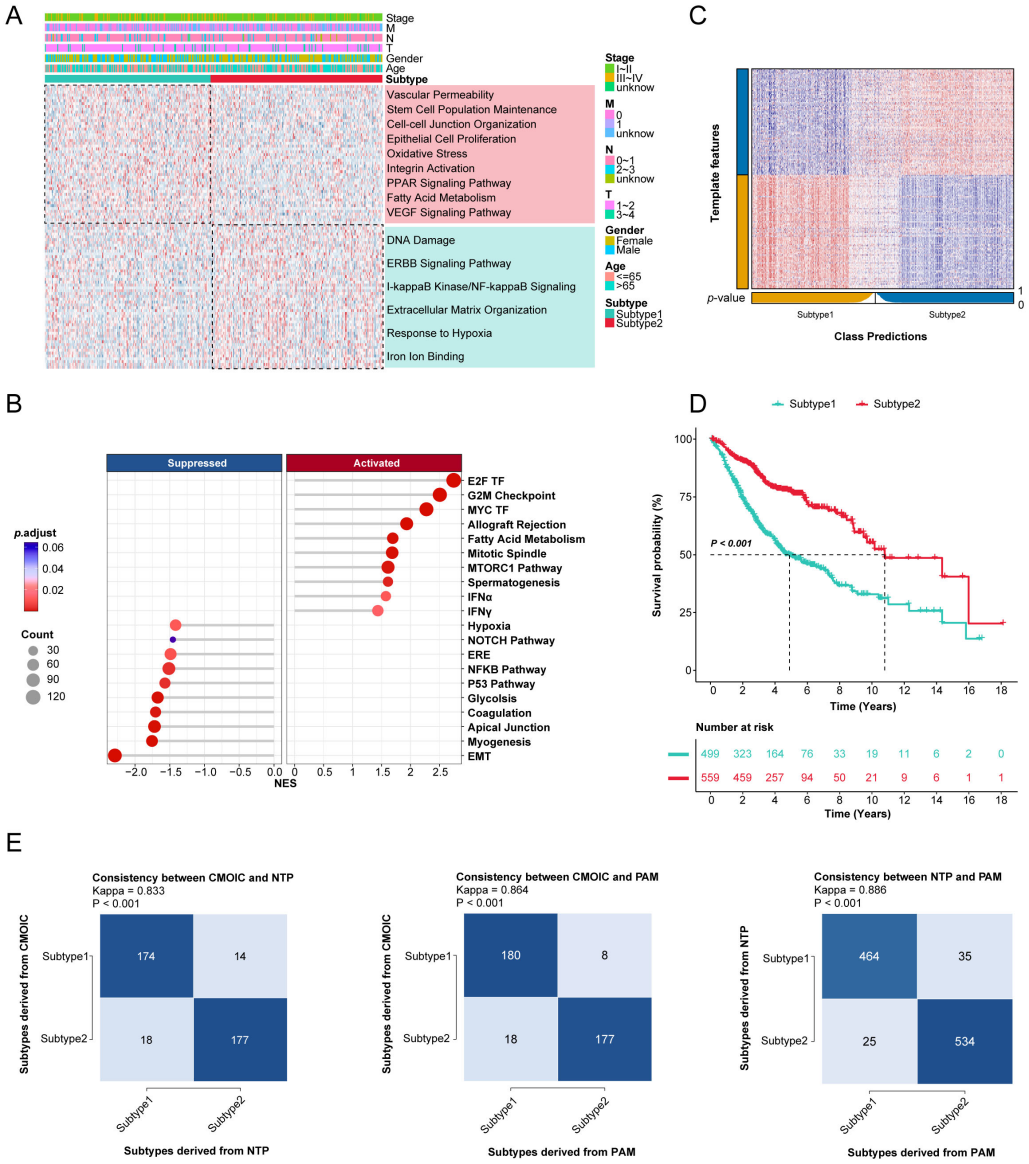
Gene enrichment analysis and validation of novel consensus subtypes in LUAD. **(A)** The GO and KEGG enrichment analyses of two consensus subtypes. **(B)** GSEA enrichment results of two consensus subtypes for hallmark repository. TF: transcription factor; MTORC: mechanistic target of rapamycin complex; IFN: interferon; ERE: estrogen response early; EMT: epithelial mesenchymal transition. **(C)** Validation of consensus subtypes in the nearest template of the integrated external validation cohort (n=1058). **(D)** Survival analysis of consensus subtypes in the integrated external validation cohort (n=1058). **(E)** The consistency of consensus subtypes with NTP, consensus subtypes with PAM, and NTP with PAM in external validation cohort (n=1058).

Currently, most molecular subtyping of LUAD relies on molecular features that correlate with specific biological functions. Therefore, we investigated the different molecular features of the two novel subtypes by conducting differential gene expression analysis and gene set enrichment analyses with GO, KEGG, and GSEA categories in the TCGA-LUAD cohort (**Figures 2A, B**, **Supplementary Table 1**). Interestingly, key biological processes and pathways, such as vascular permeability, the VEGF signaling pathway, and epithelial cell proliferation were significantly enriched in subtype 1, while subtype 2 characterized by a heightened response to hypoxia, indicative of a hypoxic tumor microenvironment.

To further validate this classification, we selected 100 upregulated genes from each subtype as classifiers and confirmed their predictive capacity across multiple external datasets (**Supplementary Table 2**). The external validation cohort consisted of 1,058 samples from six different GEO datasets (**Supplementary Table 3**). The Nearest Template Prediction (NTP) method was utilized to categorize samples in validation datasets according to predefined consensus subtypes (**Figure 2C**), aligning with initial findings that subtype 1 exhibited poorer prognoses compared to subtype 2 (**Figure 2D**). The consistency of these consensus subtypes was also evaluated with NTP and partitioning around medoids (PAM) algorithms (**Figure 2E**).

### Assessment of the TME in novel consensus molecular subtypes of LUAD

**Figure 3:**
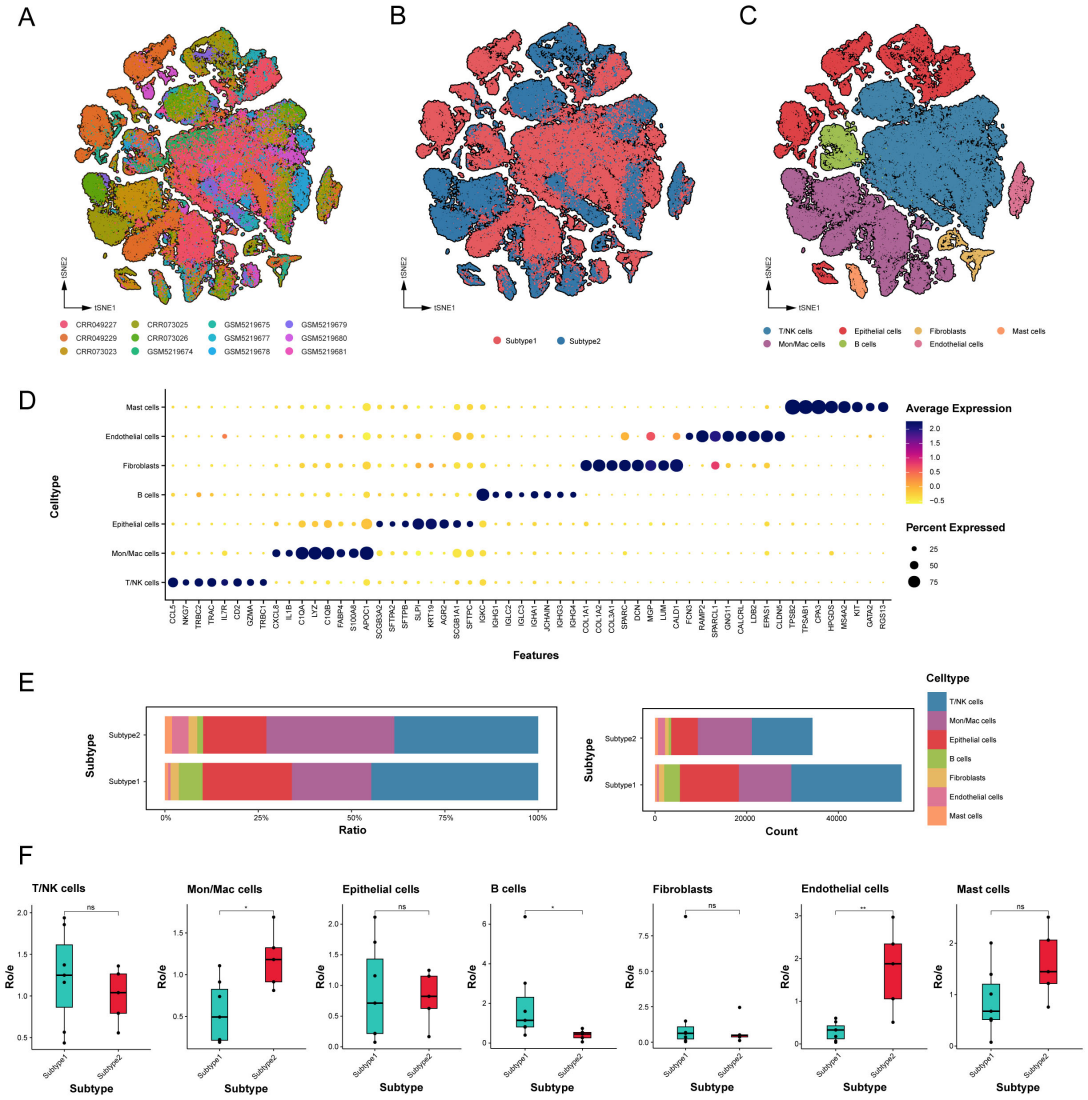
Global landscape and cell types in novel subtypes of LUAD samples. **(A-C)** tSNE projection of 88,100 profiled cells from 12 LUAD samples that have been identified into two novel subtypes, and color-coded by different samples, subtypes and major cell lineages. **(D)** Dot plot of mean expression of top 8 marker genes for 7 major lineages. **(E)** Relative proportion and count of cell major lineages for each subtype. **(F)** Tissue preference of each cell major lineages that were quantified by the calculation of the ratio of observed cell numbers to expected cell numbers (Ro/e) determined by a chi-square test. Black dots represent different samples. ns. *p* > 0.05; * *p* < 0.05; ** *p* < 0.01; two-sided Student’s t test.

The integration of 12 tumor samples from LUAD patients across two independent datasets facilitated comprehensive bioinformatics analyses of the tumor microenvironment differences between these subtypes (**Supplementary Figures 1A, B**). Eighty-eight thousand, one hundred single cells were clustered into seven lineages and annotated based on canonical marker genes: T/NK cells, B cells, Mon/Mac cells (monocytes and macrophages), mast cells, fibroblasts, epithelial cells, and endothelial cells (**Figures 3A-C**). All cell types underwent enrichment analysis of DEGs to evaluate annotation accuracy (**Figure 3D**, **Supplementary Table 4**).

The relative proportions and absolute counts of various cell types within the TME differed significantly between the two subtypes of LUAD (**Figure 3E**). Epithelial cells, T/NK cells, and Mon/Mac cells predominated in both subtypes, with subtype 1 exhibiting higher proportions of epithelial cells, T/NK cells, and B cells, while subtype 2 evidenced a higher prevalence of endothelial cells, mast cells, and Mon/Mac cells. To assess subtype distribution preference, the ratio of observed cell numbers to expected counts (Ro/e) was computed (**Figure 3F**, **Supplementary Table 5**), highlighting the significant differences in distribution among major cell types.

### Adverse immune microenvironment in the poor-prognosis LUAD subtype

**Figure 4:**
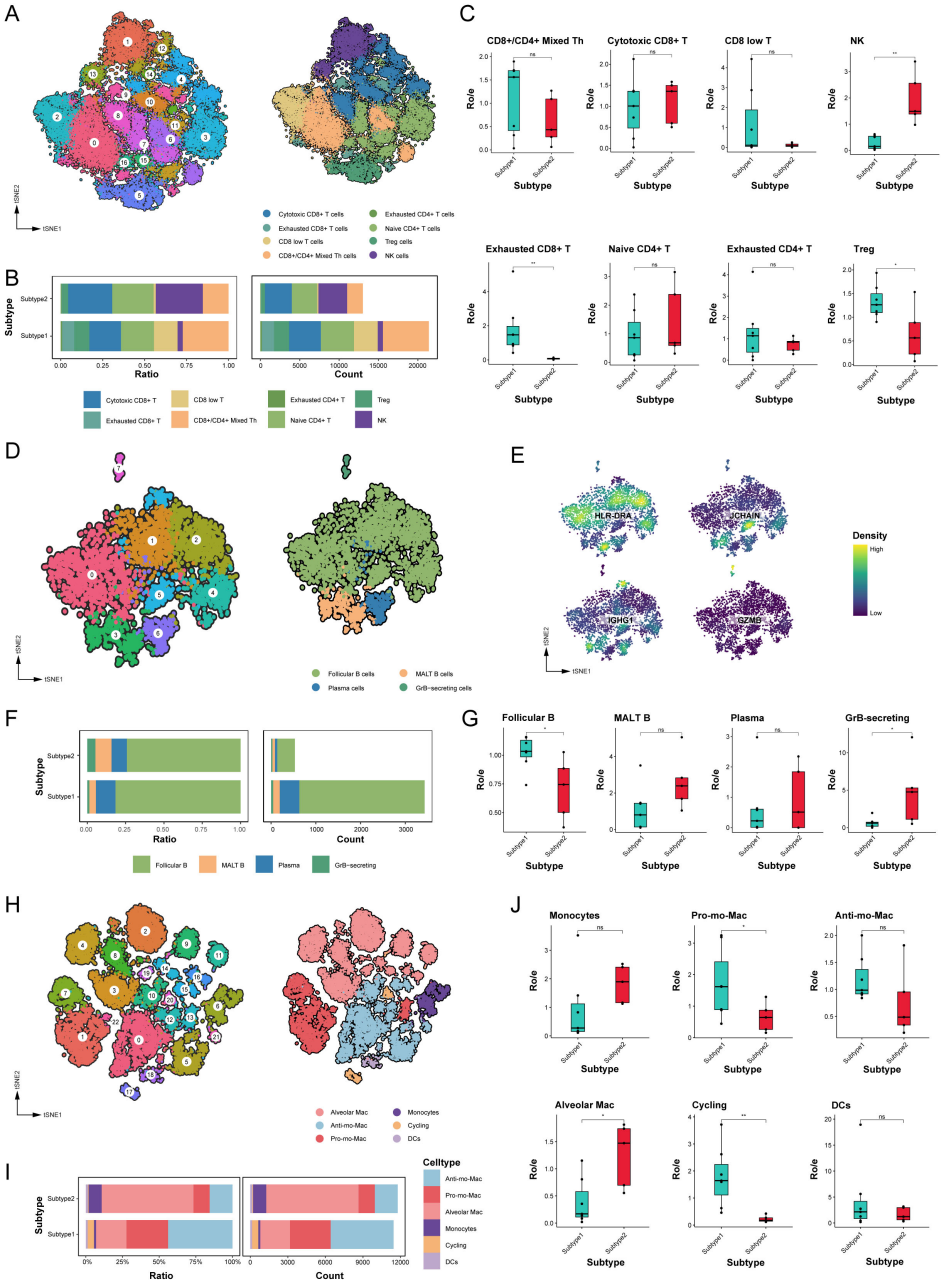
The immune microenvironment varied significantly between different molecular subtypes. **(A)** tSNE plot of T and NK cells, color-coded by clusters and cell subsets as indicated. Tfh: T follicular helper; Th: T helper; Treg: Regulatory T. **(B)** Relative proportion and cell count of T and NK cells subsets from samples of each novel subtype. **(C)** Tissue preference of T and NK cells subsets. **(D)** tSNE plot of B cells, color-coded by clusters and cell subsets as indicated. GrB, granzyme B; MALT: mucosa-associated lymphoid tissue. **(E)** tSNE color-coded by expression of canonical marker genes for each B cells subset. **(F)** Relative proportion and cell count of B cells subsets from samples of each novel subtype. **(G)** Tissue preference of B cells subsets. **(H)** tSNE plot of myeloid cells, color-coded by clusters and cell subsets as indicated. Pro-: Pro-inflammatory; Anti-: Anti-inflammatory. **(I)** Relative proportion and cell count of myeloid cells subsets from samples of each novel subtype. **(J)** Tissue preference of myeloid cells subsets. ns. *p* > 0.05; * *p* < 0.05; ** *p* < 0.01; two-sided Student’s t test.

T and NK cells account for a significant proportion of TME cell populations and are essential mediators of anti-tumor immunity. We analyzed the T/NK cell populations, isolating a total of 37,275 cells from the T/NK cluster, reclassifying them into 17 distinct clusters based on functional states and DEGs (**Figure 4A**, **Supplementary Figure 2A**). Noteworthy disparities were observed, with subtype 1 exhibiting a reduction in NK cell proportions and an increase in exhausted CD8^+^ T and Treg cells compared to subtype 2 (**Supplementary Table 6**). The marked persistence of exhausted CD8^+^ T and Treg cells in subtype 1 suggests a mechanism contributing to immune evasion during tumor progression (**Figures 4B, C**).

We also assessed B cell populations, which mediate anti-tumor immune responses associated with prolonged patient survival (29, 30). Our analysis revealed eight clusters diverging into four differentiation states among 3,964 B cells (**Figures 4D, E**). Follicular B cells constituted the largest proportion among all LUAD samples, with a significantly higher abundance in subtype 1 than subtype 2 (**Figures 4F, G**). Furthermore, subtype 2 displayed greater numbers of granzyme B-secreting GC B cells, which can enhance cytotoxicity and function as alternatives to T cells (**Supplementary Table 6**).

Myeloid cells play a crucial role in maintaining lung tissue homeostasis and regulating inflammatory responses. As shown in **Figure 4H**, our analysis categorized 23,220 myeloid cells into 23 subclusters, identifying subclusters as monocytes, macrophages, and dendritic cells (DCs). Alveolar macrophages, possessing important homeostatic functions, displayed heightened expression of specific genes, such as MARCO, MCEMP1, and FABP4 genes. Different to tissue-resident macrophages, Mo-Macs were recruited from circulating monocytes and exhibited distinct phenotypes, including pro-inflammatory Mo-Macs (highly expressed IL1B and CXCL8) and anti-inflammatory Mo-Macs (high expression of APOE, CD163, and C1QB genes). Comparative analysis indicated that subtype 1 exhibited a higher abundance of pro-inflammatory Mo-Macs and proliferating myeloid cells, whereas subtype 2 had a higher concentration of alveolar macrophages (**Figures 4I, J**, **Supplementary Figure 2B**, **Supplementary Table 6**).

### Cell-to-cell communication analyses in novel subtypes of LUAD

**Figure 5:**
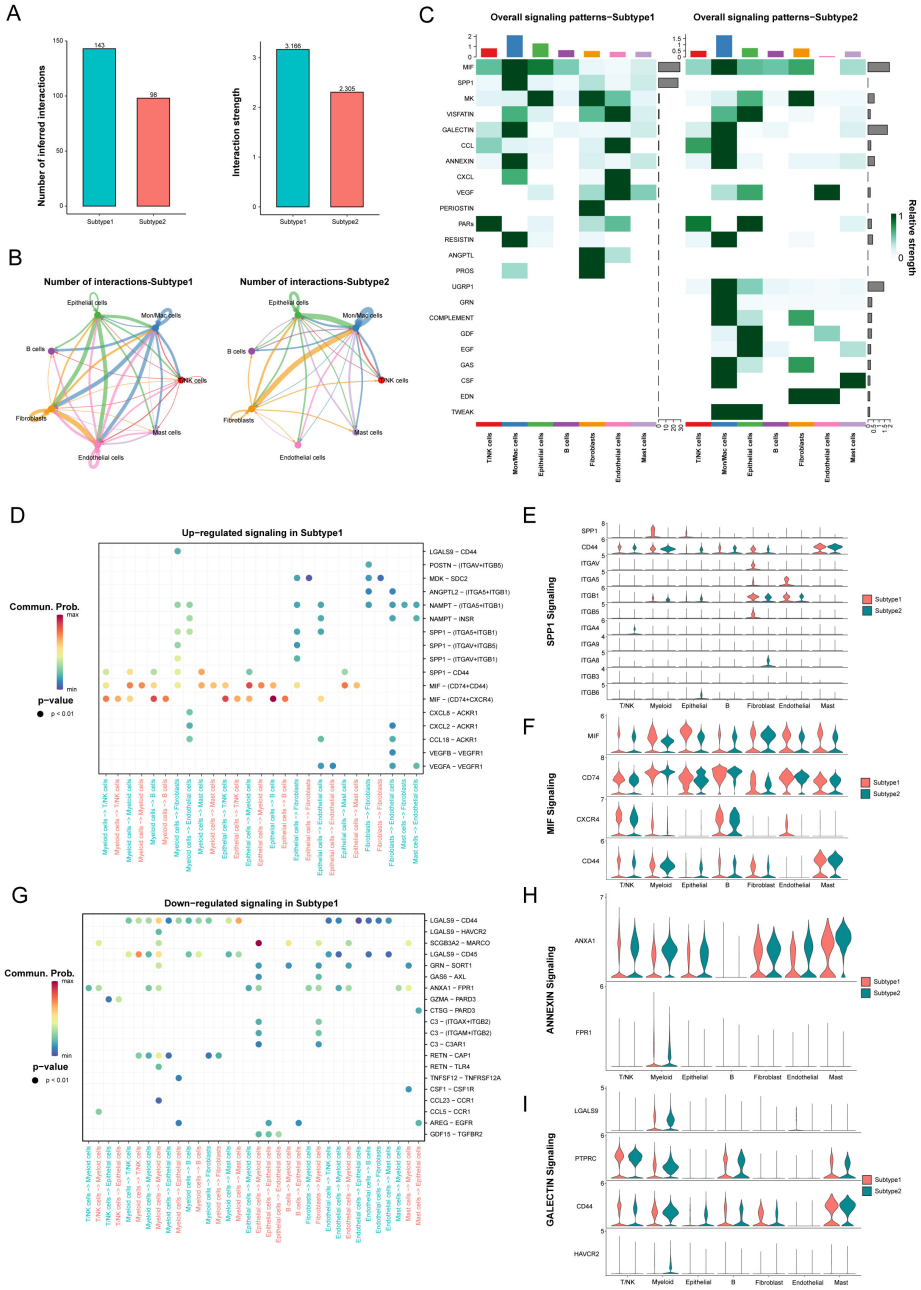
The difference of signaling pathways between two novel subtypes in LUAD. **(A)** The number of inferred interactions and the interaction strength between different molecular subtypes. **(B)** The number of inferred interactions for each subtype. **(C)** The overall signaling of each cell population between different subtypes. **(D-I)** Identification of up- and down-regulated signaling in the Subtype 1 through the comparison of communication probabilities mediated by ligand-receptor pairs in all cell populations.

The influence of cell-cell communication has been r recognized as crucial on the tumor immune microenvironment. To clarify intercellular communications differences between these two novel subtypes, we utilized the “CellChat” package to analyze networks of communication signals from scRNA-Seq data. Many significant ligand–receptor pairs were detected among cell types, with subtype 1, exhibiting substantially higher interaction frequencies and strengths (**Figures 5A, B**). Moreover, the endothelial cells contribute most to the outgoing or incoming signals in the number of inferred interactions, while the fibroblasts contribute most to the outgoing and the B cells contribute most to the incoming signals in the interaction strength. However, the communication between fibroblasts and myeloid cells achieved the highest relative values. Then, we overviewed the outgoing and incoming signaling in these subtypes (**Supplementary Figure 3A**).

The main incoming signals and outgoing signals in subtype 1 were MIF and SPP1 signaling, and SPP1, GALECTIN and UGRP1 signaling in subtype 2 (**Figure 5C**, **Supplementary Figures S3B, C**). Furthermore, we identified altered ligand-receptor pairs among these cell types by comparing their communication probabilities between different subtypes. Results showed that MIF signaling, such as MIF-(CD74+CXCR4), MIF-(CD74+CD44), and SPP1 signaling, especially SPP1-CD44, were increased in myeloid cells and epithelial cells to their receivers in the subtype 1 compared to the subtype 2 (**Figures 5D-F**). However, ANNEXIN signaling, such as ANXA1-FPR1, and GALECTIN signaling, such as LGALS9-CD44 and LGALS9-CD45 were decreased from myeloid cells and endothelial cells to their receivers in the subtype 1 (**Figures 5G-I**). Our analysis identified specific signaling pathways, including MIF and SPP1 signaling in subtype 1, which were noted for their implications in tumor progression and immunosuppression.

### Development of a multi-Omics machine learning-driven prognostic signature in LUAD

**Figure 6:**
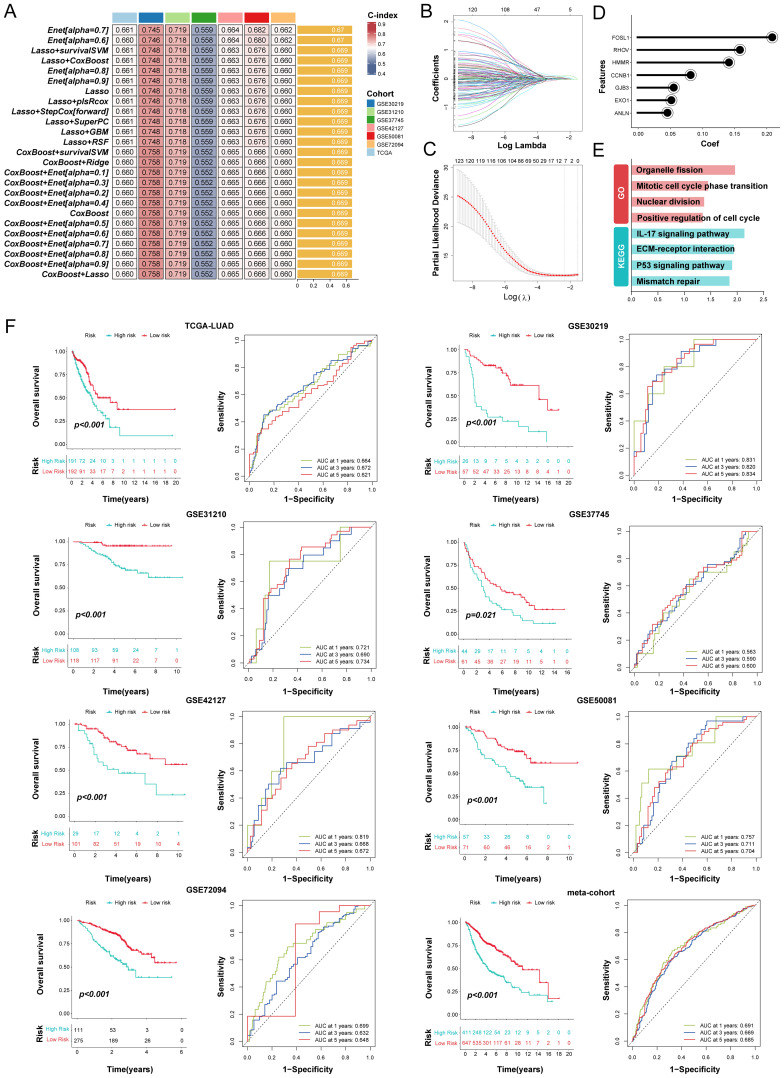
Integration of multiple machine learning algorithms developed a prognostic signature in LUAD patients. **(A)** The top 25 kinds of prediction models based on a comprehensive computational framework and then the C-index of each model was calculated through training dataset and all validation datasets. **(B, C)** Coefficients of 7 prognosis-related genes selected by Enet [alpha = 0.7] regression. The regularization parameter λ is used to select covariates. **(D)** Lollipop plots displaying the coefficients of the MO-MLPS genes. **(E)** GO and KEGG term enrichment results of the MO-MLPS gene set. **(F)** Survival analysis and ROC curves for OS at 1-, 3-, and 5-years for all LUAD patients classified into high-risk and low-risk groups based on the MO-MLPS. The analysis includes data from the TCGA-LUAD (n = 383), GSE30219 (n = 83), GSE31210 (n = 226), GSE37745 (n = 105), GSE42127 (n = 130), GSE50081 (n = 128), GSE72094 (n = 386) cohorts, and a meta-cohort (n = 1058) for validation.

Through univariate Cox regression, a total of 123 prognosis related genes were filtered from 200 specifically upregulated for each LUAD subtype in the TCGA-LUAD (as training cohort) and 6 GEO datasets (as validation cohort). We integrated these candidate genes within an ensemble machine-learning framework to construct the MO-MLPS (**Figure 6A**, **Supplementary Figure 4**). Our predictions revealed that the Enet [alpha=0.7] algorithm yielded the highest average C-index (0.67), showcasing far superior predictive capabilities compared to alternative methodologies in both training and validation cohorts (**Figures 6B-D**, **Supplementary Tables 7, 8**). Hence, the seven genes MO-MLPS constructed via Enet [alpha=0.7] algorithm was identified as the final risk signature: risk score = 0.21003 × FOSL1 + 0.05394 × EXO1 + 0.05671 × GJB3 + 0.14348 × HMMR + 0.08324 × CCNB1 + 0.04620 × ANLN + 0.15915 × RHOV. The results of GO and KEGG for the seven genes in the risk signature enrichment in biological processes related to the cell cycle, nuclear division, and organelle fission, as well as pathways of mismatch repair and P53 signaling pathway (**Figures 6E**).

The resulting MO-MLPS, defined by the risk score equation, subdivided patients into high- and low-risk groups with markedly differing clinical outcomes. As illustrated in **Figure 6F**, patients with high-risk score had significantly poorer clinical outcomes compared to those with low-risk score in the training and validation datasets. Furthermore, the meta-cohort dataset that merged all validation patients showed the same trend. Subsequently, the discrimination of our signature were assessed via ROC analysis, with 1-, 3-, and 5-year AUCs of 0.664, 0.672, and 0.621 in TCGA-LUAD; 0.831, 0.820, and 0.834 in GSE30219; 0.721, 0.690, and 0.734 in GSE31210; 0.563, 0.590, and 0.600 in GSE37745; 0.819, 0.668, and 0.672 in GSE50081; 0.757, 0.711, and 0.704 in GSE50081; 0.699, 0.632, and 0.648 in GSE72094; 0.691, 0.669, and 0.685 in meta-cohort, respectively.

### Evaluation of the MO-MLPS performance

Given the proliferation of transcriptome-based prognostic signatures reported in contemporary literature, we performed a systematic review to compare the predictive efficacy of the MO-MLPS against previously published signatures. Exclusions were applied for signatures relying on miRNA and lncRNA due to dataset limitations. In total, 49 distinct signatures were analyzed (**Supplementary Table 9**), with the MO-MLPS demonstrating superior predictive performance, especially within the meta-cohort (**Supplementary Figure 5**, **Supplementary Table 10**). Furthermore, those signatures performed better than the MO-MLPS presumably because in their own training set or a few internal validation datasets, while performed weakly in other datasets.

**Figure 7:**
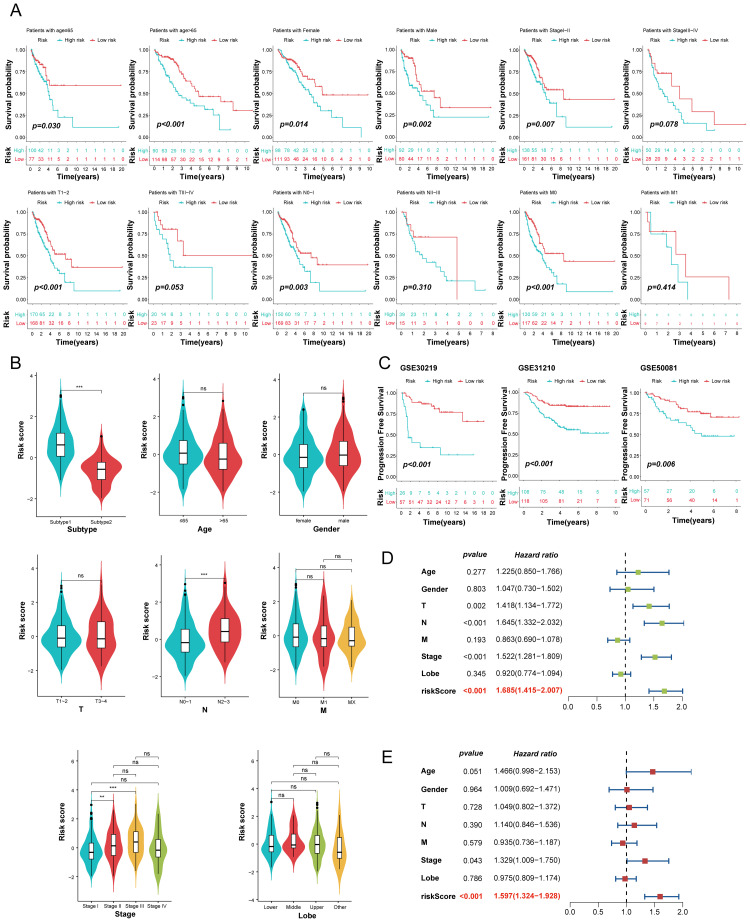
Evaluation of the MO-MLPS predictive power for the prognosis of LUAD patients. **(A)** Survival comparison analysis in different clinical subgroup of TCGA-LUAD cohort, including age, gender, AJCC stage and clinic stage. **(B)** Violin plots illustrated the relationship among the MO-MLPS high-risk and low-risk score in different clinical subgroup in TCGA-LUAD cohort, including subtype, age, gender, AJCC stage, clinic stage and lung lobe. **(C)** Kaplan-Meier analysis of progression-free survival of LUAD patients between the MO-MLPS high-risk and low-risk groups. **(D, E)** The univariate and multivariable Cox regression analysis results of the MO-MLPS in TCGA-LUAD cohort. Data are presented as mean ± 95% confidence interval [CI]. ns. *p* > 0.05; * *p* < 0.05; ** *p* < 0.01; *** *p* < 0.001; two-sided Student’s t test was used between two groups; one-way ANOVA test was used among multiple groups.

To further evaluate the prognostic value of the MO-MLPS in LUAD patients, a stratification analysis was performed within different subgroups. The MO-MLPS demonstrated robust performance in predicting OS across different subgroups, including LUAD patients aged ≤ 65 and > 65, both male and female subgroups, those classified within Stage I~II, tumor stage 1~2 and 3~4, as well as nodal stage 0~1 and metastatic stage 0 (**Figure 7A**). There was no significant difference between different subgroups stratified by age, gender, AJCC-T, AJCC-M and Lobe but significant between subgroups stratified by AJCC-N and Stage I~III (**Figure 7B**). Then, the predictive value of the MO-MLPS for progression-free survival of LUAD patients was assessed in GSE30219, GSE31210 and GSE50081 cohorts. According to the Kaplan-Meier curve, LUAD patients with a high-risk score demonstrated a worse progression-free survival than those with a low-risk score (**Figure 7C**). Furthermore, univariate and multivariate Cox regression analyses were performed to verify the risk score of the MO-MLPS as an independent prognostic biomarker in the TCGA datasets (**Figures 7D, E**). In univariate regression analysis, the MO-MLPS risk score, age, AJCC-T, AJCC-N and Stage were associated with patient OS significantly. Multivariate cox regression analysis identified that the MO-MLPS risk score and Stage were significant independent risk factors for the OS. Notably, in both univariate and multivariate Cox regression analyses, the hazard ratio associated with the risk score exceeded that of conventional clinical indicators, which might suggest that the risk score may have a comparatively greater impact on prognosis of LUAD patients.

### Immune characteristics related to the MO-MLPS

**Figure 8:**
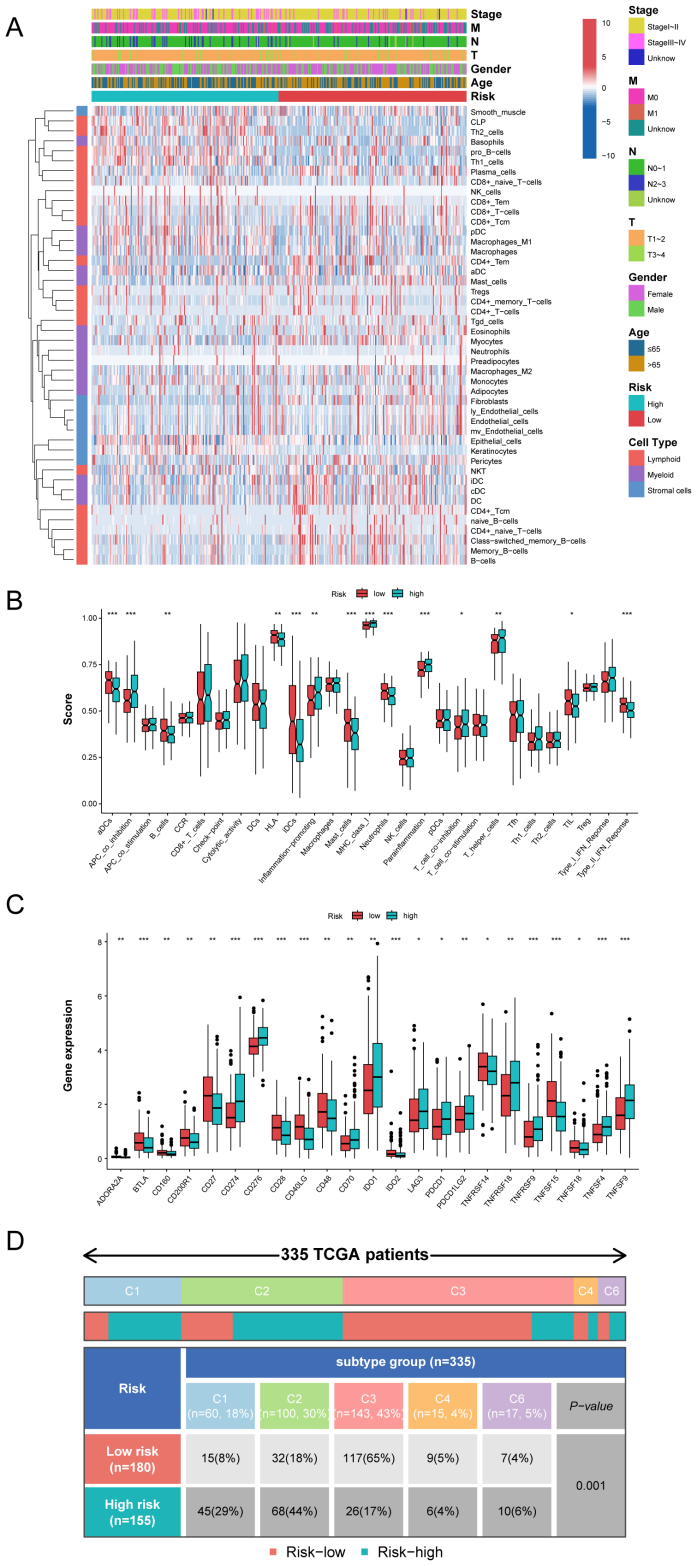
The immune microenvironment landscape in different the MO-MLPS risk group. **(A)** The relationship between the MO-MLPS risk score and immune microenvironment infiltrations in TCGA-LUAD dataset. **(B, C)** The distribution of 28 immune-related cell types and immune checkpoint genes between the MO-MLPS high-risk and low-risk patients. **(D)** 335 patients in the TCGA-LUAD cohort were accordingly divided into 5 different immune subtypes and each immune subtype were statistically different between the MO-MLPS high- and low-risk subgroups (*P* < 0.001).

Employing the xCell deconvolution algorithm in Immuno-Oncology Biological Research (IOBR) R package, we performed immune cell abundance analysis and observed immune cell infiltration levels of TME in LUAD (**Figure 8A**). Notably, most effector and cytotoxic T-lymphoid (CD4+ naive T, CD4+ Tcm, CD4+ Tem and CD8+ T cells), mature B-lymphoid (Class switched memory B, B and plasma cells) and effector myeloid cell lines (aDC, cDC, iDC, and myocytes cells) were significantly higher in the MO-MLPS low-risk patients than in high-risk patients, which is suggestive of a state of immune activation (**Supplementary Figure 6**). These results suggested an immunoactivity phenotype among low-risk patients, with heightened levels of effector immune cells and cytotoxic T-lymphoid populations. Conversely, high-risk patients exhibited an immunosuppressive profile with reduced immune cell infiltration, suggesting a cold tumor environment.

To evaluate the characteristics and tumor microenvironment among patients with different the MO-MLPS risk score, a total of 28 immune infiltration scores were assessed between high- and low-risk subgroups via the ssGSEA method. The result showed that patients were categorized into high-risk group had significantly higher score of APC co-inhibition, inflammation-promoting, MHC class I, para-inflammation and T helper cells than low-risk group, while the score of DCs, B cells, HLA, IDCs, mast cells, neutrophils and type II IFN response in the low-risk group were higher than that in the high-risk group (**Figure 8B**). We further investigated the implications of the MO-MLPS risk scores on immune checkpoint expression. According to the result, we found that a variety of classical immune checkpoint molecules, including ADORA2A, BTLA, CD160, CD200R1, CD27, CD28, CD40LG, CD48, IDO2, TNFRSF14, TNFSF15 and TNFSF18 were more highly expressed in the MO-MLPS low-risk group but the expression of CD274, CD276, CD70, IDO1, LAG3, PDCD1, PDCD1LG2, TNFRSF18, TNFRSF9, TNFSF4 and TNFSF9 were higher in high-risk group (**Figure 8C**). Furthermore, 335 patients in the TCGA-LUAD cohort were divided into 5 different immune subtypes. In the low-risk MO-MLPS group, the majority of patients (65%) were classified under the C3 immune subtype, whereas in the high-risk MO-MLPS group, the predominant immune subtype was C2 (44%). And patients of C4 and C6 subtypes were accounted for nearly equal proportion between low and high risk (**Figure 8D**). In addition, Tumor Immune Dysfunction and Exclusion (TIDE) scores, a robust metric for predicting patient responses to immune checkpoint inhibitors (ICIs), were calculated to evaluate potential differences in immunotherapy response between the high-risk and low-risk groups identified by the MO-MLPS. Nevertheless, no significant differences were observed in TIDE scores, microsatellite instability, dysfunction, exclusion, myeloid-derived suppressor cells, and cancer-associated fibroblasts between the MO-MLPS high-risk and low-risk groups (**Supplementary Figure 7**).

### Effects of ANLN gene knockdown on LUAD cells behavior

**Figure 9:**
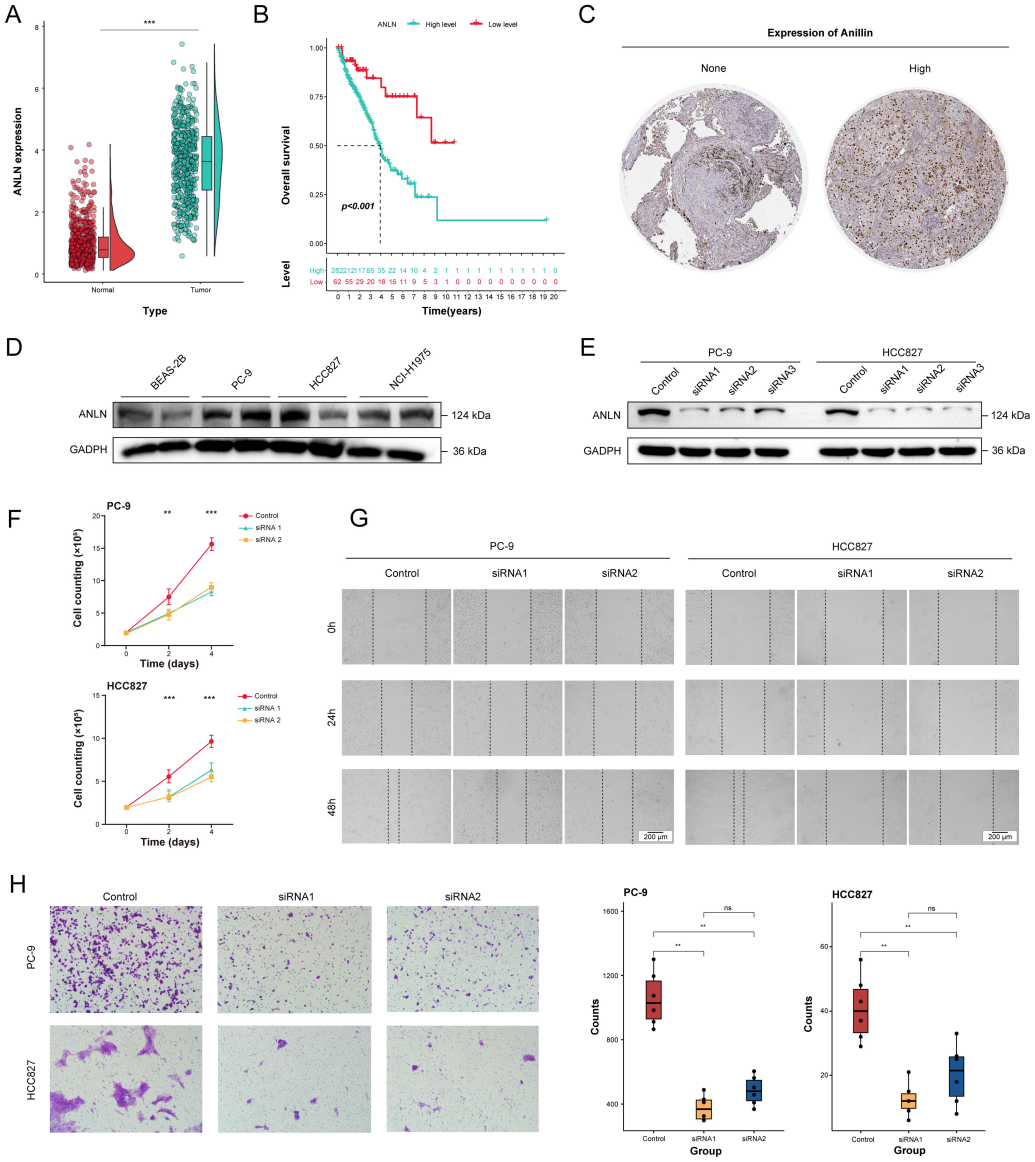
The decrease of ANLN expression affected the proliferation and migration ability of human LUAD cells. **(A)** Differential expression analysis for ANLN between tumor tissues (n = 541) and normal tissues (n = 637) through integrating TCGA and GTEx database. **(B)** The Kaplan-Meier survival curves of the high- and low-expression ANLN groups in LUAD patients. **(C)** Representative Immunohistochemistry images showing the protein expressions of anillin. **(D)** The expression levels of anillin in BEAS-2B, PC-9, HCC827 and NCI-H1975 cell lines. **(E)** The effect of ANLN knockdown on anillin expression was measured by western blot analysis. **(F)** Cell proliferation evaluated by direct cell counting for ANLN knockdown in LUAD cells. **(G, H)** Representative images and statistical boxplots of migration ability of LUAD cell with ANLN knockdown assessed by scratch assay and transwell migration assay. ns. *p* > 0.05; * *p* < 0.05; ** *p* < 0.01; *** *p* < 0.001; two-sided Student’s t test was used between two groups; one-way ANOVA test was used among multiple groups.

Given the robust performance of our signature in predicting the prognosis of LUAD patients, we next investigated the possibility of these seven genes as therapeutic targets for LUAD. We integrated LUAD samples from TCGA database and healthy samples from the Genotype-Tissue Expression (GTEx) database to identify mRNA expression characteristics of these genes. The results showed that the transcription levels of ANLN was highly expressed in most tumor samples and associated with prognosis of LUAD patients (**Figures 9A, B**). Then, the protein expression levels of anillin, encoded by the ANLN gene, in LUAD tumor and para-cancerous tissues were explored via the Human Protein Atlas (HPA) database. Expression of anillin showed that the protein mainly accumulated in the nucleus of LUAD cells (**Figure 9C**).

To elucidate the potential effects of ANLN on biological features of LUAD cells, the expression pattern of anillin in the different LUAD cell lines was assessed through western blotting. The results showed that expression of anillin in carcinoma cell lines (PC-9, HCC827 and NCI-H1975) was highly relative to healthy lung bronchial epithelial cell (BEAS-2B) (**Figure 9D**). Then, PC-9 and HCC827 with higher levels of anillin were adopted for subsequent studies. We knockdown anillin expression significantly in the PC-9 and HCC827 cell lines through transfection with siRNAs (**Figure 9E**). After 48 hours transfection, the number of proliferating cells significantly decreased with the suppression of ANLN (**Figure 9F**). Given the anillin is an actin binding protein and involved in cytoskeletal stability. Therefore, scratch wound healing and transwell migration assay was were performed in PC-9 and HCC827 with ANLN silencing markedly to evaluate the impacts of it on cell migration. The result demonstrated that cell migration ability was decreased significantly upon ANLN knockdown, as compared to cells transfected with the negative control (**Figures 9G, H**).

### The domain negative anillin protein expression improved the sensitivity of LUAD cells to docetaxel treatment

The above *in vitro* study indicated that the ANLN gene or anillin protein could serve as potential targets for therapeutic intervention. However, there were no drugs or small molecule inhibitors directly inhibiting ANLN activity and the approach of targeting siRNA is limited in current clinical utilization, which would be the challenges for the clinical application of ANLN. Anillin is a unique scaffolding protein, which regulates major cytoskeletal structures, such as microtubules, actin filaments and septin polymers (31). The N-terminal region of anillin contains binding sites for actin and other cytoskeletal regulators, whereas the C-terminal region contains a pleckstrin homology (PH) domain that facilitates anillin interacting with the equatorial membrane (32). Therefore, we engineered a domain-negative anillin (dnANLN) protein, the C-terminally truncated anillin mutant, that loses its ability to bind cytoskeletal regulators but still retained the PH domain to interact with furrows.

**Figure 10:**
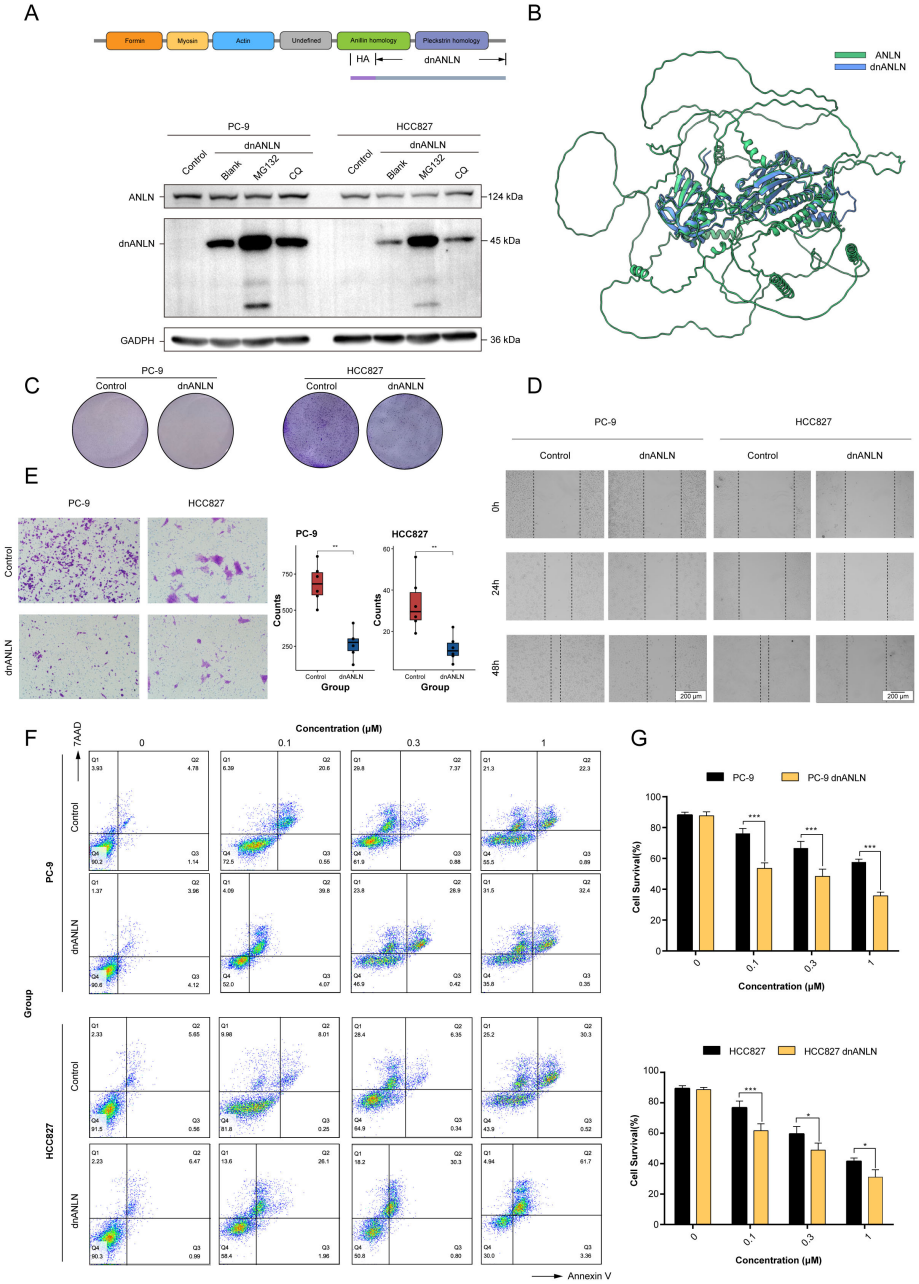
The expression of recombinant dnANLN protein improved the sensitivity of LUAD cells to docetaxel treatment. **(A)** Schematic illustration of the dnANLN protein. And the levels of intracellular dnANLN protein expression were determined by western blot. The addition of MG132 affected the protein expression levels of dnANLN. CQ: chloroquine; dnANLN: domain negative anillin. **(B)** A tertiary structure prediction of dnANLN protein was generated using homology modeling method via the AlphaFold3 platform. **(C)** The colony formation assay was performed to assess the effect of dnANLN protein expression on colony-forming ability. **(D, E)** The evaluation of migration ability affected by the intracellular expression of dnANLN protein through scratch assay and transwell migration assay in LUAD cells. **(F, G)** The effect of dnANLN protein expression on the viability of LUAD cells subjected to docetaxel treatment. Cell viability of PC-9 and HCC827 were detected by flow cytometry using an Annexin V/7AAD assay. ns. *p* > 0.05; * *p* < 0.05; ** *p* < 0.01; *** *p* < 0.001; two-sided Student’s t test.

The results showed that the molecular mass of domain negative anillin protein was approximately 45 kDa. Notably, the addition of the proteasome inhibitor MG132 or the lysosomal inhibitor chloroquine increased the protein expression level of dnANLN, but the effect of the former was more pronounced (**Figure 10A**). This suggested that dnANLN might mainly degraded via the ubiquitin-proteasome pathway. To investigate if the truncation affected the structure of the anillin protein, a tertiary structure prediction was performed through AlphaFold3 (https://alphafoldserver.com/). It appeared that the truncation did not affect the overall structure of anillin (**Figure 10B**). Then, a colony formation assay was conducted to evaluate the impact of dnANLN on colony-forming capacity and cellular viability. The result demonstrated that the expression of dnANLN declined the number of colony formation and decreased cell viability (**Figure 10C**). Furthermore, results from scratch wound healing and transwell migration assay indicated that the expression of dnANLN dramatically inhibited LUAD cell *in vitro* migration (**Figures 10D, E**). Docetaxel is a commonly chemotherapeutic drug for the treatment of NSCLC and acts through stabilizing microtubules and prevent their depolymerization. Notably, the expression of dnANLN markedly increased docetaxel-induced cytotoxicity in PC-9 and HCC827 cell lines, which suggested that domain negative anillin protein could improve the drug sensitivity of LUAD cells to docetaxel treatment (**Figures 10F, G**).

## Discussion

Gene expression is a complex and multifactorial process that involves diverse mechanisms and interactions among numerous components, including mutation, methylation, histone modifications, and post-transcriptional RNA modification (33, 34). Therefore, comprehensive integration of multi-omics data from patients can provide deeper insights into disease-specific regulatory mechanisms. However, current research predominantly focuses on single-omics approaches (28). Furthermore, the selection of clustering methods for omics is mainly influenced by individual preferences, which consequently exacerbates the limitations of specific methods with expansion of the scope of use. To address these limitations, two novel prognostic LUAD subtypes with distinct characteristics were identified via integrating the latest 10 clustering algorithms, which may have significant potential for accurate stratified treatment of LUAD patients. These two novel subtypes showed consistent stability across multiple cohorts and revealed significant difference in overall survival. In most previous studies, the assessment of immune cell infiltration among different subtypes have primarily relied on bulk-tissue immune scoring algorithms (29, 35–37). However, with the rapid advancement of scRNA-Seq techniques in recent years, it has been possible to quantitatively characterize cell types at a single-cell resolution. In this study, we systematically investigated differences in immune infiltration and intercellular communication between two novel LUAD subtypes at the single-cell resolution level.

Our analysis revealed a significant upregulation of SPP1 and MIF expression in both myeloid and epithelial cells within the poor-prognosis subtype. Specifically, these myeloid and epithelial cells interact with T/NK cells, additional myeloid cells, B cells, fibroblasts, and mast cells through three distinct ligand-receptor axes: SPP1-CD44, MIF-(CD74+CD44) or MIF-(CD74+CXCR4) signaling pathway. SPP1 encodes the protein secreted phosphoprotein 1, which functions as a chemokine that regulates immune cell differentiation and proliferation (38). It has been reported that elevated levels of SPP1 in tumor cells are correlated with a poor prognosis in NSCLC (39). On the one hand, MIF can activate tumor cell proliferation contributing to tumor progression. On the other hand, MIF can enhance the immunosuppressive microenvironment by increasing the abundance of MDSCs within tumors (40).

At present, high-throughput sequencing technology has been widely applied for clinical diagnosis and treatment as well as in the investigation of the pathogenic mechanisms underlying various diseases. Moreover, complete and high-quality transcriptional information serve as critical biomarkers for prognostic stratification and therapeutic strategy optimization. Machine learning algorithms should be an effective and popular tool to analysis RNA-seq data. We identified specifically upregulated genes in each novel LUAD subtypes and developed a novel prognostic prediction signature in the one TCGA dataset and six GEO datasets using 100 algorithm combinations. Finally, the Enet algorithm [α = 0.7] was selected and defined as the MO-MLPS, based on the average C-index from training and multiple validation datasets. Consistently across all cohorts, the high-risk group identified by the MO-MLPS exhibited significantly poorer survival outcomes. Then, the MO-MLPS indicated significant prognostic value across majority of cohorts in comparison to other published signatures. And this signature was identified as an independent risk factor for LUAD patients in both univariate and multivariate Cox regression. Notably, one of the external validation sets, GSE37745, showing an AUC value of less than 0.6. By comparison, we found that LUAD patients with advanced stage account for a high proportion in the GSE37745 dataset. Given that advanced cancer harbors a high level of heterogeneity of cells, patients with advanced cancer may be were more heterogeneous compared to patients with non-advanced cancer in LUAD. According to the results, the MO-MLPS had a high a high prognostic predictive accuracy which is robust and stable in different datasets, indicating a great prospect for future clinical transformation and application.

In this study, the MO-MLPS was composed of 7 prognosis-related genes (FOSL1, EXO1, GJB3, HMMR, CCNB1, ANLN, RHOV) identified in LUAD patients. Most of these genes have well- established roles in LUAD tumorigenesis, particularly in modulating proliferation, invasion, and metastatic cascades. First, FOS-like antigen 1 (FOSL1) is a very important member of the FOS family, which responsible for encoding leucine zipper proteins that dimerize with the JUN family proteins, forming the AP-1 transcription factor complex (41). Recent studies have shown that the FOSL1 may be a potential prognostic marker and target for human lung adenocarcinoma with KRAS mutations (41, 42). Then, Exonuclease 1 (EXO1) plays a pivotal role in maintaining genomic stability through coordinating dual activities: RNase H and 5’ to 3’ exonuclease functions. These activities are essential for DNA repair, regulation of cell cycle checkpoints, and the dynamics of telomeres (43). It has been reported that the increased expression of EXO1 is correlated with larger tumor size, increased tumor metastasis, suppressed immune cell infiltration and poor overall survival in LUAD patients (44–46). The protein encoded by Gap Junction Protein Beta (GJB3) is a component of gap junctions, connexin 31, which has been indicated that highly expressed in the tissues of LUAD patients and positively correlated with LUAD stages. And the expression of GJB3 was also associated with a poor prognosis in LUAD (47, 48). Furthermore, Hyaluronan Mediated Motility Receptor (HMMR), also named CD168, encodes protein forming a complex with BRCA1 and BRCA2 (49). Previous studies reported that the level of HMMR affected cell cycle, DNA replication and cell metabolism in LUAD tissues (50). And the expression of HMMR in LUAD was greater than that in the health, which could increase the progression or recurrence of LUAD patients (51). Cyclin B1 (CCNB1) acts as the primary regulator of the G2/M transition, with its expression reaching a peak during mitotic entry (52). It has been demonstrated that the overexpression of CCNB1 is closely associated with increased cell proliferation, migration and tumorigenesis in LUAD cells (53–55). Anillin (ANLN) plays a critical role in scaffolding actomyosin networks, which are essential for cytokinesis and mechanical stress adaptation (56). The expression levels of ANLN have been reported elevated in LUAD cells, and LUAD patients with higher levels of ANLN had a relatively poor prognosis (56–59). Ras Homolog Family Member V (RHOV) is a constituent of the Ras superfamily of small GTPases. The overexpression of RHOV has been implicated in the enhancement of proliferation, migration, invasion and epithelial-to-mesenchymal transition of LUAD cells (60, 61). Furthermore, elevated expression levels of RHOV may be indicative of reduced overall survival in LUAD patients (62).

To strengthen the robustness of the MO-MLPS, our study utilized a multi-cohort validation framework, including the TCGA-LUAD training cohort and six independent GEO validation cohorts, encompassing a total of 1,441 LUAD patients. The total sample size across all cohorts ensures sufficient statistical power for detecting clinically meaningful survival differences. Moreover, we observed substantial event rates in all cohorts, which meet the recommended thresholds for survival analysis power. Furthermore, the reproducibility of the MO-MLPS across six GEO datasets and a meta-cohort minimizes the risk of false-positive results. The pooled C-index and AUCs across cohorts indicate the robust discriminatory power of the MO-MLPS, which is corroborated by its superior performance compared to nearly 49 existing prognostic signatures. However, it is important to acknowledge that smaller validation cohorts or subgroups may diminish statistical power. Nonetheless, the consistency of significance levels across all datasets alleviates this concern. Moreover, the MO-MLPS demonstrated a large effect size in both univariate and multivariate analyses, thereby reducing the likelihood of type II errors. Notably, the HR associated with risk scores were found to be greater than those of conventional clinical indicators, suggesting that the observed survival differences are unlikely attributable to random variation. The combination of large event numbers, multi-cohort validation, and biologically meaningful effect sizes underscores the reliability of our survival analyses, even in stratified subgroups. Future prospective studies with pre-specified power calculations will be necessary to further validate these findings.

Given that the impact of tumor microenvironment on the prognosis of patients, we further investigated the discrepancy of immune cell infiltration in different the MO-MLPS risk group. The results indicated that insufficient infiltration of immune cells and impaired immune regulation exacerbate the “immune desert” phenotype in the MO-MLPS high risk group. The proportion of major cells that participate in cancer cell killing and tumor elimination, including CD4+ T cells, CD8+ T cells, mature B cells, monocytes and dendritic cells, were lower in the MO-MLPS high risk LUAD patients than those with the MO-MLPS low risk. Although elevated infiltration levels of Th1 cells could inhibit tumor growth, this protective effect might be counterbalanced by increased Th2 cells. Moreover, according to the tumor immunotyping in TCGA, we found that the proportion of patients with C3 and C4 subtypes in the MO-MLPS low risk patients was higher than that in the MO-MLPS high risk, while the proportion of patients with C1, C2 and C6 tumors in the MO-MLPS low risk patients was lower. In recent years, the checkpoint inhibitor immunotherapy has been one of the most significant treatments in LUAD patients. Therefore, analysis of the expression levels of checkpoint genes in the MO-MLPS high risk and low risk groups was performed. Intriguingly, the results indicated that the checkpoint gene expression levels of CD274 and PDCD1, which can encode PD-L1 and PD-1 protein inducing the suppression of anti-tumor immunity, were higher in high-risk patients than in low-2risk patients. This suggests that our MO-MLPS would be used to evaluate the expression of immune checkpoint genes, and LUAD patients with high-risk score may benefit more from anti-PD-L1 or PD-1 immunotherapy through relieving immune cells from the suppressed tumor microenvironment. TIDE is a computational framework designed to model and quantify tumor immune evasion mechanisms, which are critical determinants of cancer progression and immunotherapy response. However, no significant differences were observed between the high- and low-risk groups based on the MO-MLPS. This lack of differentiation may be due to the fact that clinical responses to immunotherapy are influenced by a complex interplay of factors, including tumor mutational burden, neoantigen presentation, myeloid-derived suppressor cell infiltration, and gut microbiome composition. These unmeasured variables might obscure the predictive value of checkpoint expression alone. Furthermore, while TIDE scores primarily reflect the baseline immune evasion potential, the dynamic evolution of checkpoint expression during disease progression or treatment might be closely associated with eventual therapeutic outcomes. In addition, although TIDE remains a valuable computational tool, its predictive accuracy varies across different cancer types and may not fully capture the biological complexity of certain soft tissue sarcomas. Therefore, clinical validation using real-world immunotherapy response data is necessary to draw definitive conclusions.

Uncontrolled cell division and reproduction is considered one of the hallmark characteristics of cancer (63). A lot of widely utilized clinical chemotherapeutic drugs have been designed to target this hallmark in order to inhibit the rapid proliferation of cancer cells. To optimize treatment strategies, it is critical to identify suitable candidates that are overexpressed in cancer cells and are associated with phase-specific cell cycle functions, thereby maximizing the therapeutic index. In the signature, we noticed that the ANLN gene, which encodes an actin-binding protein involved in cell growth, division and migration, have been identified as a potential target for the development of novel therapeutic strategies and the design of new pharmacological agents for the treatment of LUAD. ANLN was significantly upregulated in adenocarcinoma cells compared with healthy lung epithelial cells, and related to the progression of LUAD patients (58). The cause of the observed cell proliferation suppression through ANLN gene depletion may be multiple. The most direct reason for this may be decreased levels anillin affected the formation or the shrinkage degree of cleavage furrow, which is the requisite element of cell division, and drive the physical separation of one cell into two cells (64). Other possible reasons may be through pyroptosis activation or the suppression of PI3K-AKT pathway (56, 58). The results of scratch assay and transwell migration assay indicated that knockdown of ANLN gene could obviously decelerate the cell migration. This might be due to anillin function as a “bridge” between actin and their binding sites, and knockdown of ANLN dampen the actin contraction and cytoskeletal remodeling which plays a key role in the process of cell migration. However, current strategies for targeting ANLN or anillin fall short of successful drug discovery and development. To compensate for this deficiency, we designed a dnANLN protein, which losing the ability to bind actin but still retained the PH domain to interact with cleavage furrows, playing a competitive inhibitory role in endogenous anillin protein (32). Similarly, our results indicated that the expression of dnANLN could inhibit colony formation and cell migration of LUAD cells. Furthermore, it further improved the sensitivity of LUAD cells to docetaxel treatment. These findings are both surprising and interesting. Our results opened up another avenue to development of novel therapeutic strategies for suppressing ANLN, which differs from conventional inhibitors and degraders.

However, the present study still has several limitations. Firstly, it is necessary to conduct large-scale prospective clinical studies to verify the predictive capability of the MO-MLPS. Second, the efficacy MO-MLPS in predicting the checkpoint gene expression levels in LUAD patients need to be further confirmed in real-world data. Furthermore, the preparation, purification and characterization of dnANLN recombinant protein will be pursued further in future research. In addition, the functional experiments were conducted in EGFR-mutant LUAD cell lines. Although these models provided consistent results, the lack of validation in molecularly distinct LUAD subtypes limits their broader applicability due to tumor heterogeneity. Future research should aim to expand validation efforts to additional models with varying molecular profiles, including primary cells or patient-derived organoids, to strengthen clinical relevance.

## Conclusion

To summarize, multi-omics data in 6 dimensions were integrated to characterize novel consensus molecular subtypes of LUAD. These subtypes had significant differences in molecular biological features, immune cell infiltration, and their prognosis also differed significantly. Based on feature genes of each subtype and multiple machine learning algorithms, a stable and robust prognostic signature, the MO-MLPS, was developed to assess the prognosis and recurrence of LUAD patients. Furthermore, cell proliferation and migratory capacity were significantly inhibited after ANLN knockdown in LUAD cells. The same effects were present in cells transfected with recombinant dnANLN and dnANLN improved the sensitivity of LUAD cells to docetaxel treatment. These results initially laid the foundation for developing dnANLN as a potential therapeutic strategy for treating LUAD in the future.

## Data Availability

Publicly available datasets were analyzed in this study. This data can be found here: <b>The dataset of TCGA-LUAD cohort can be obtained from The Cancer Genome Atlas Program(https://portal.gdc.cancer.gov/). All dataset of GSE72094, GSE50081, GSE42127, GSE37745, GSE31210 and GSE30219 can be downloaded from Gene Expression Omnibus (GEO) data base (https://www.ncbi.nlm.nih.gov/geo/).</b>.
