# Supplementary material for: Identification of novel molecular subtypes and construction of a prognostic signature via multi-omics analysis and machine learning in lung adenocarcinoma
Source: Front Oncol. 2025 Jul 21;15:1590216. doi: 10.3389/fonc.2025.1590216 (PMC12320504; doi:10.3389/fonc.2025.1590216)
Supplement: Supplementary file 1 [file DataSheet1.docx]

Supplementary Material

# Supplementary Figures and Tables

## Supplementary Figures


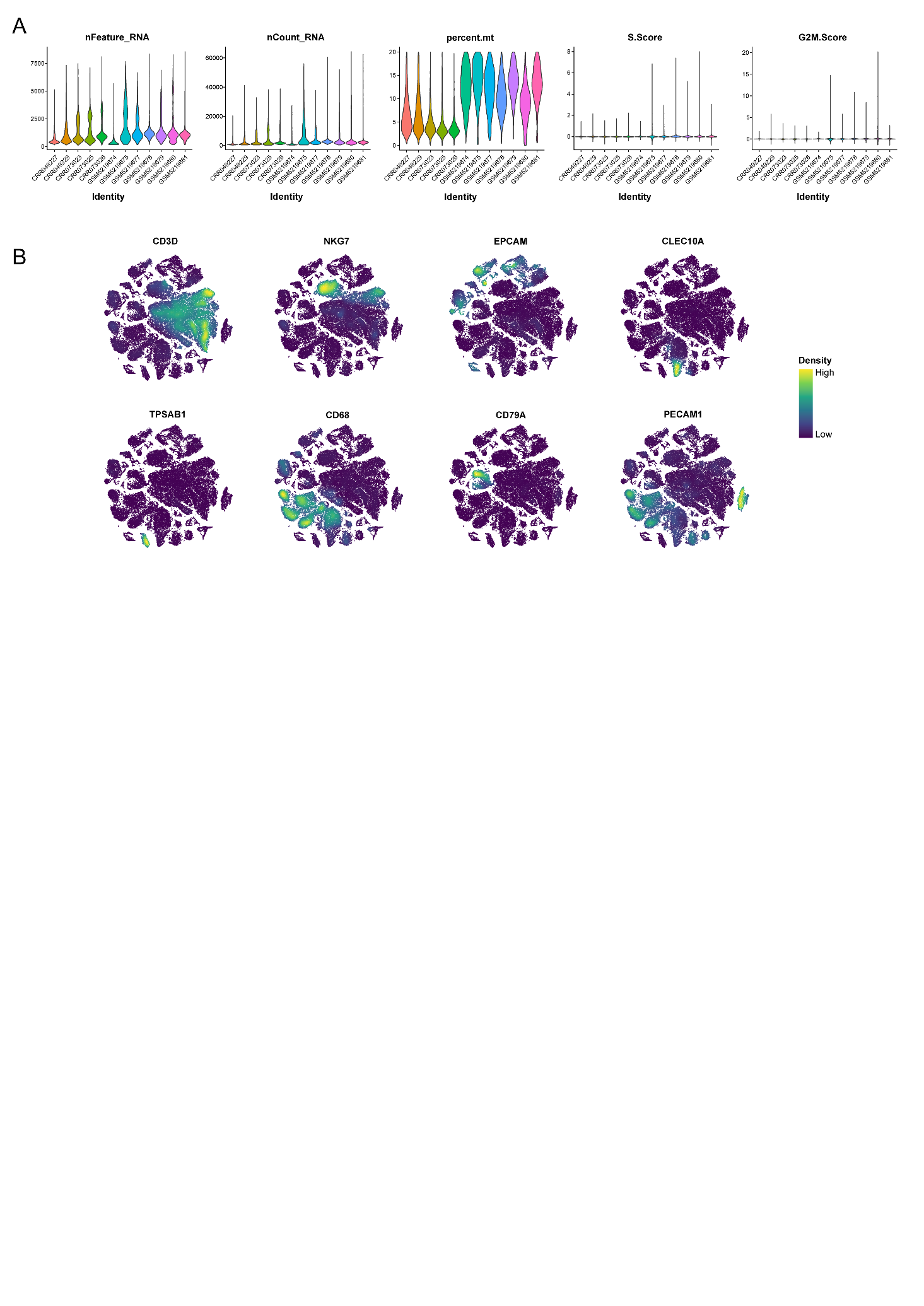
**Supplementary Figure 1. The basic parameters of all single-cell RNA-seq samples and gene signatures of distinct major cell subsets.** (A) The violin diagram of gene numbers (nFeature_RNA), sequencing depth (nCount_RNA), mitochondrial gene percentage (percent.mt), S-phase scores (S. Score) and G2/M scores (G2M. Score) in LUAD samples. (B) tSNE color-coded by expression of canonical marker genes for each major lineage.


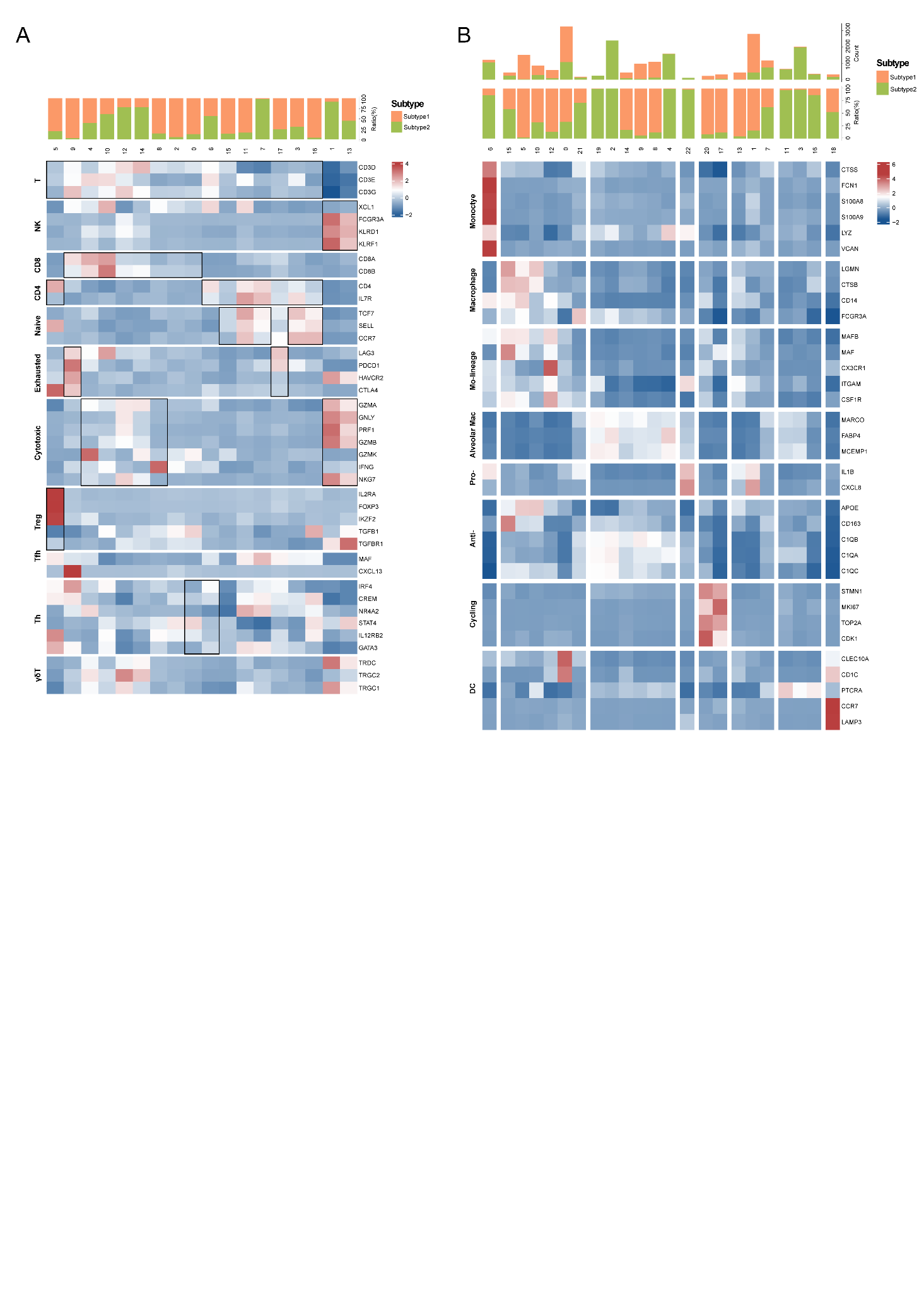
**Supplementary Figure 2. Marker gene expression heatmap of each subgroup of T/NK cells and myeloid cells.** (A) Comprehensive heatmap of selected T and NK cells marker genes across each cell cluster. (B) Comprehensive heatmap of selected myeloid cells marker genes across each cell cluster. The top annotation illustrated relative expression of selected marker genes associated with each cell subset. Mean expression of marker genes were scaled by mean-centering.


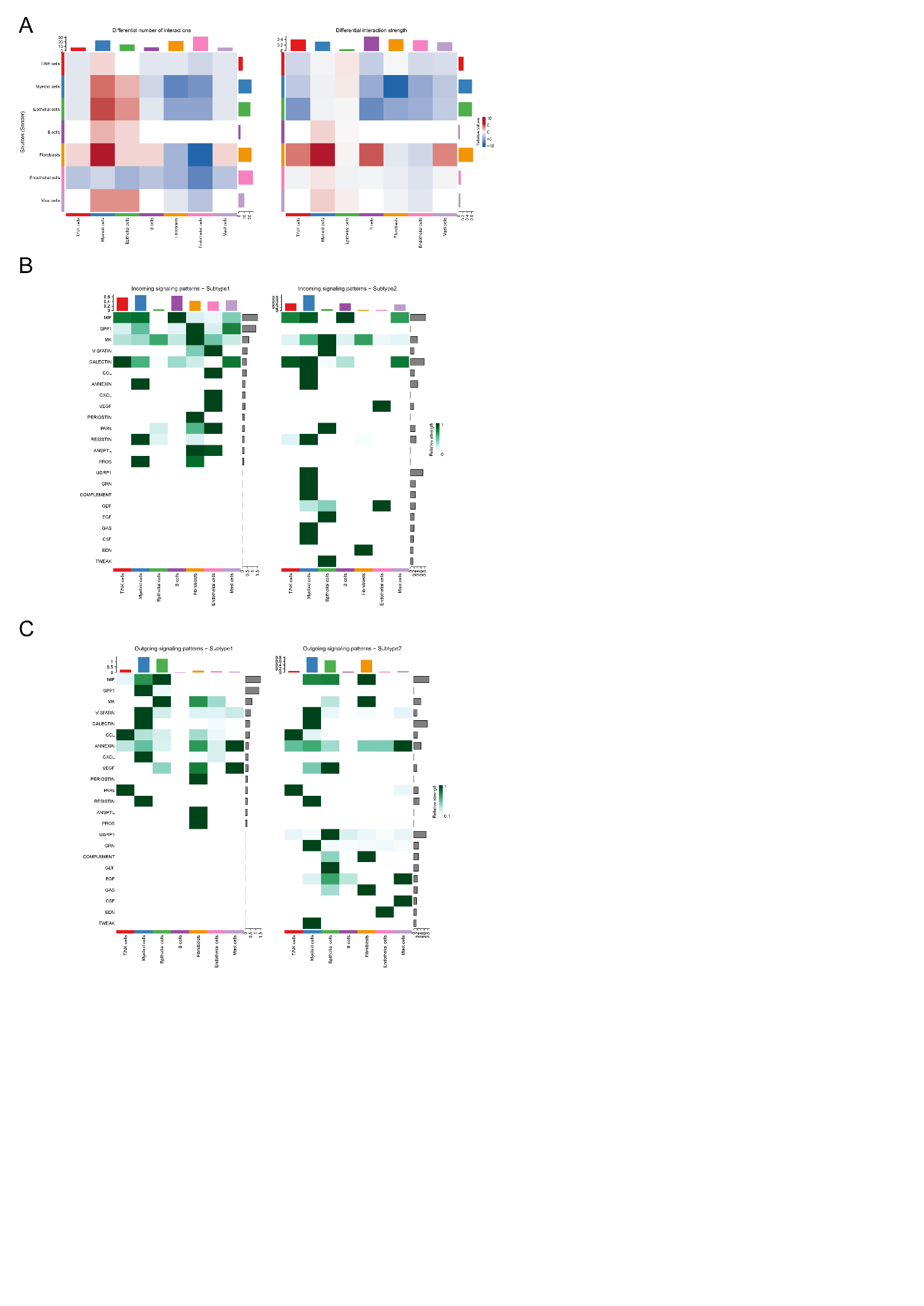


**Supplementary Figure 3. Differential interaction and signaling patterns between two novel subtypes in LUAD.** (A) Differential number of interactions and strength of each cell population. (B) & (C) The incoming and outgoing signaling patterns of each cell population between two novel subtypes.


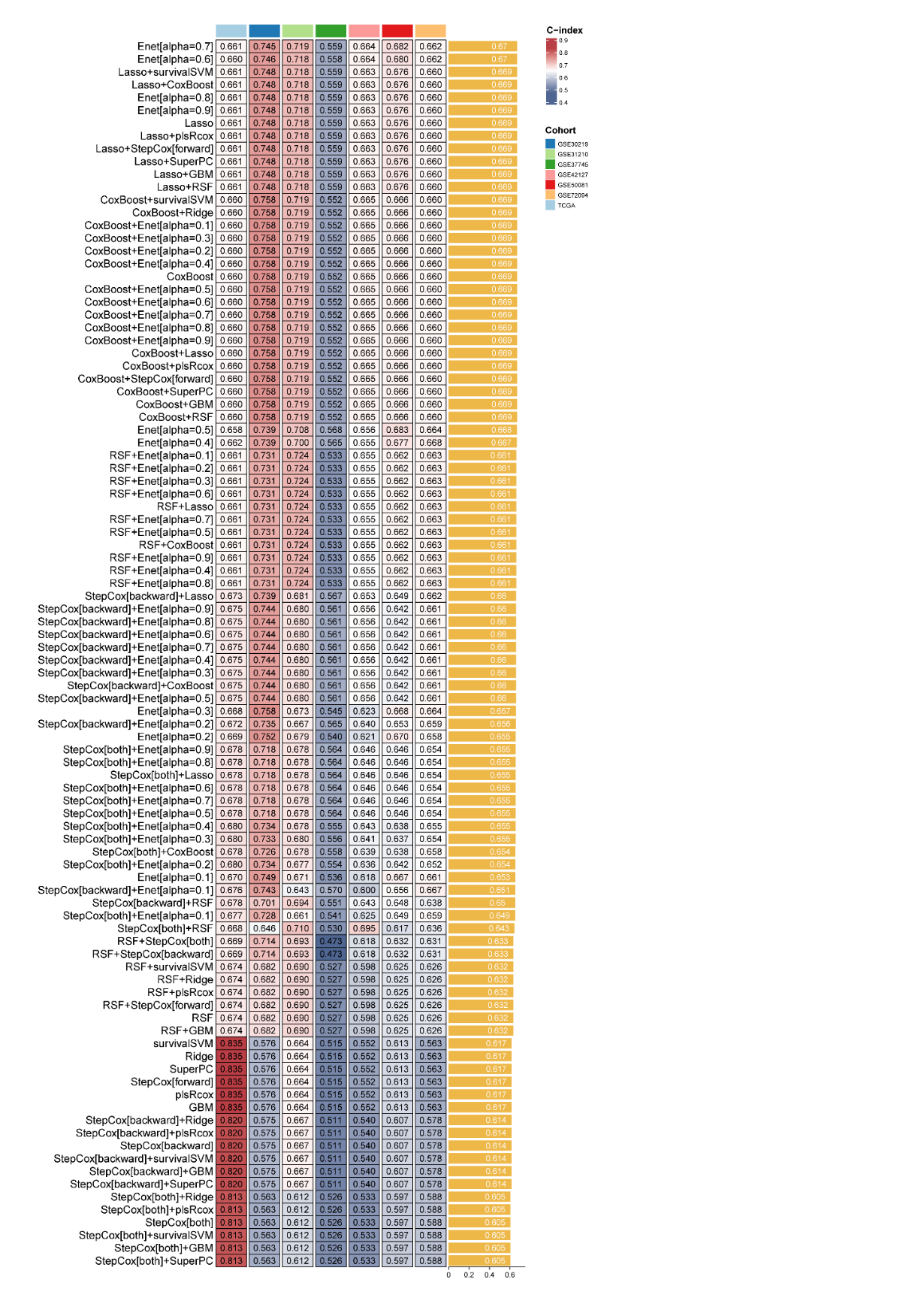


**Supplementary Figure 4. Integrated multiple machine learning algorithms-driven prognostic signature in LUAD.** A total of 100 prediction models based on a comprehensive computational framework and then the C-index of each model was calculated through training dataset and 6 validation datasets.


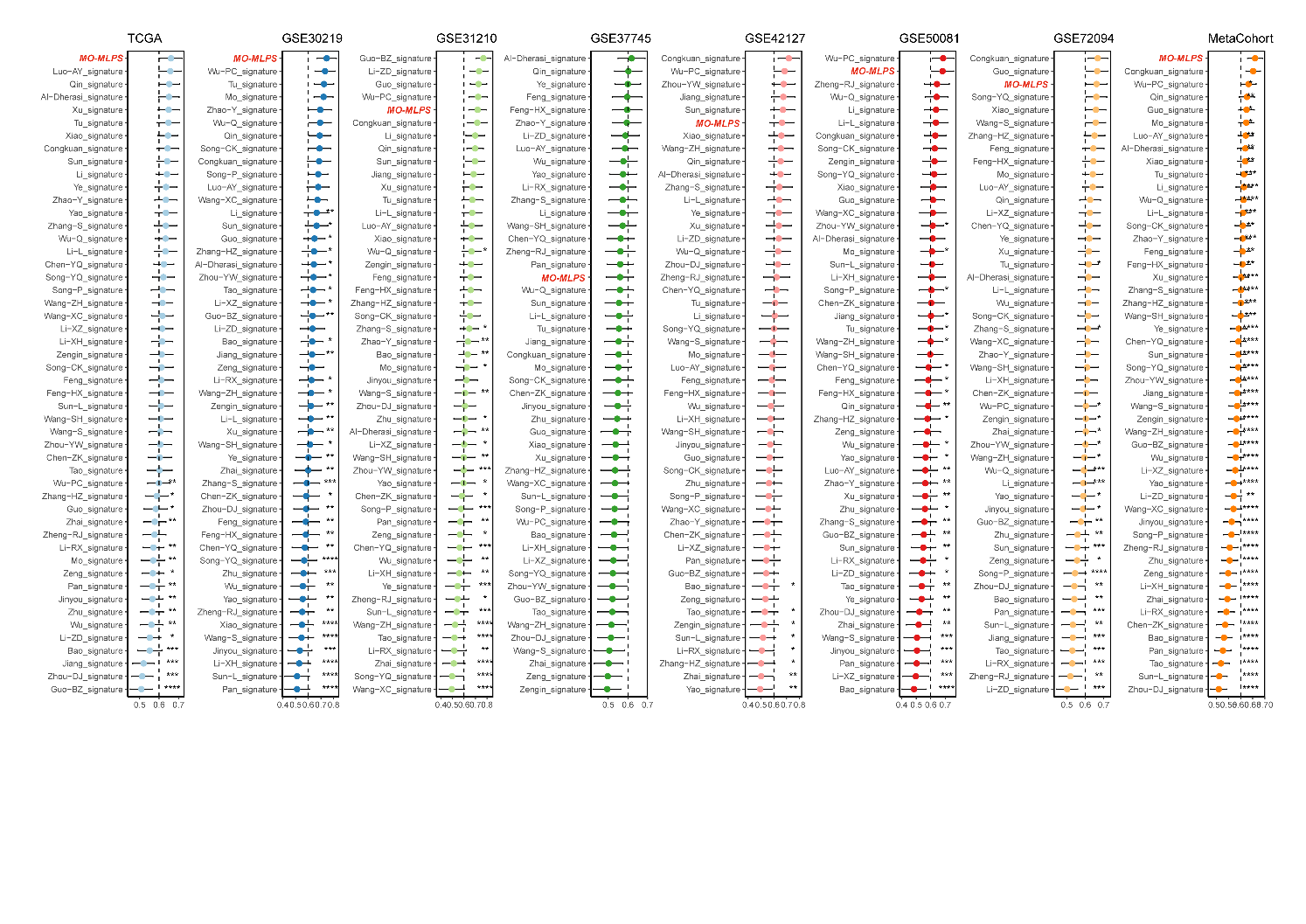
**Supplementary Figure 5. Comparison of the accuracy of the prognostic models.** Comparison of C-index between the MO-MLPS and the other 50 prognostic models that have been published in the TCGA-LUAD, GSE30219, GSE31210, GSE37745, GSE42127, GSE50081, GSE72094 cohorts and validation meta-cohort. Data are presented as mean $\pm$ 95% confidence interval [CI]. ns. *p* > 0.05; * *p* < 0.05; ** *p* < 0.01; *** *p* < 0.001.


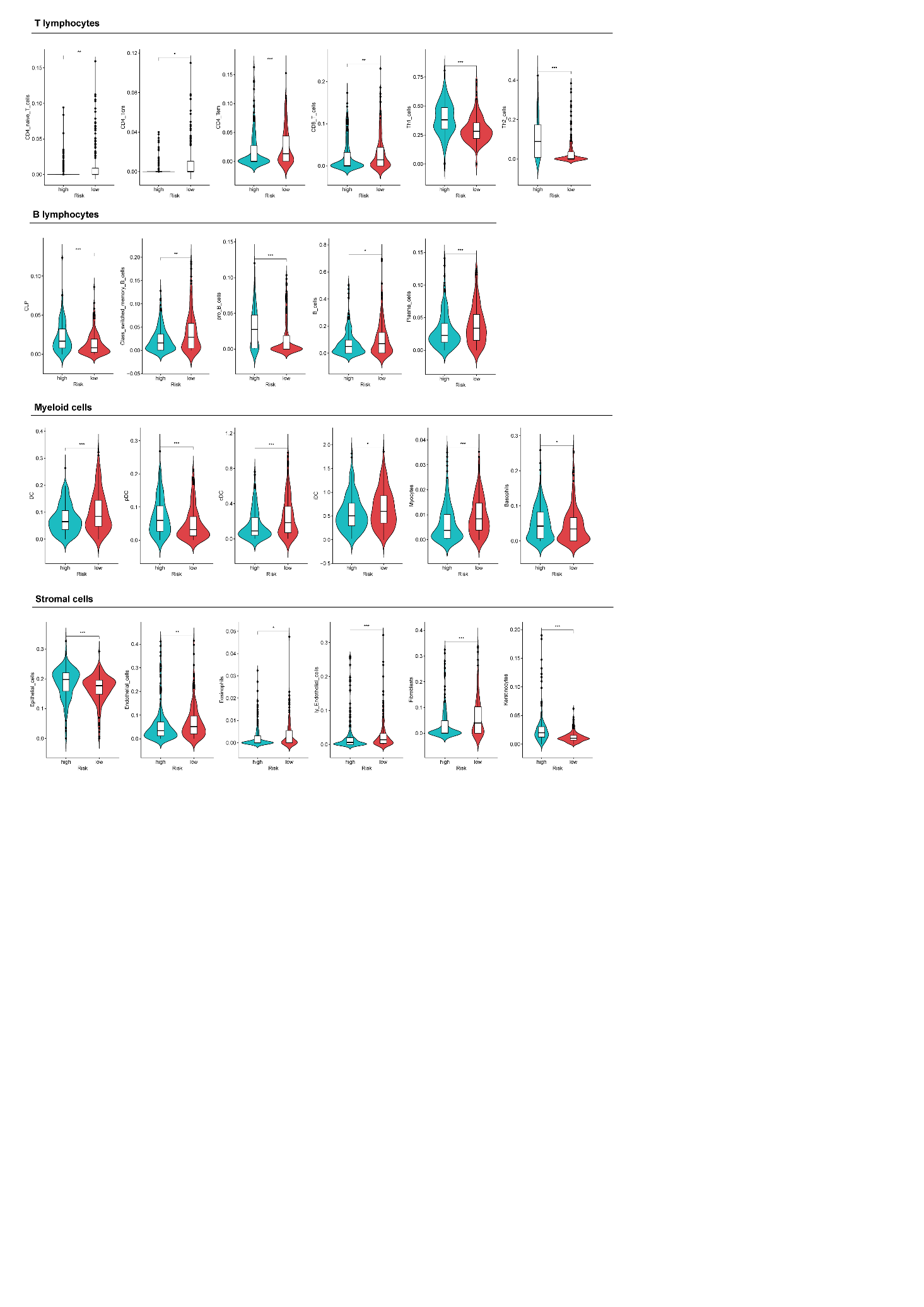


**Supplementary Figure 6. Immune microenvironment analysis in different MO-MLPS risk score groups.** The violin plots illustrated the infiltration of immune and stromal cells, demonstrating statistically significant differences between the high-risk and low-risk subgroups of MO-MLPS. ns. *p* > 0.05; * *p* < 0.05; ** *p* < 0.01; *** *p* < 0.001.


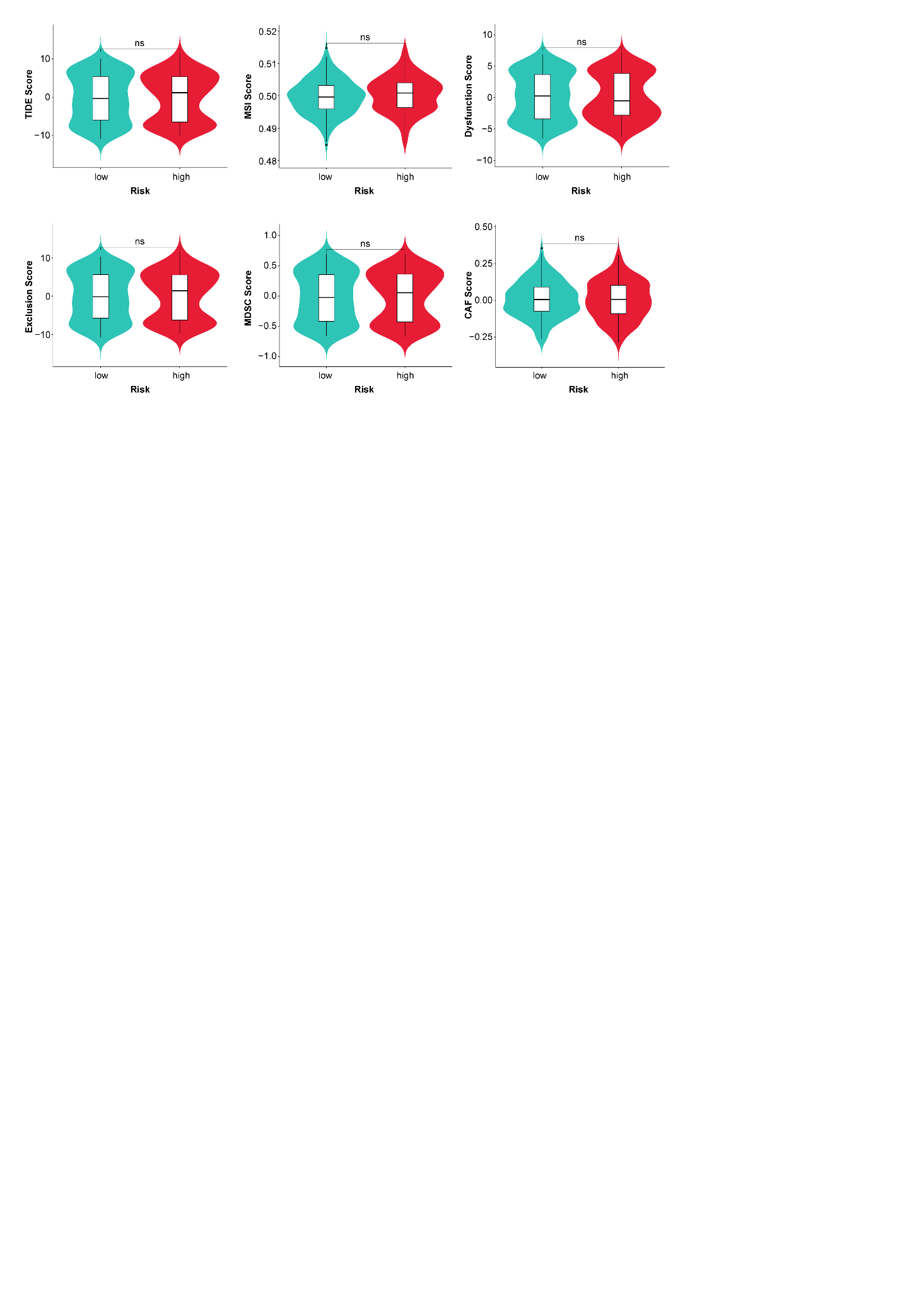
**Supplementary Figure 7. TIDE analysis in different MO-MLPS risk score groups.** Violin plots illustrated the score of TIDE, microsatellite instability (MSI), dysfunction, exclusion, myeloid-derived suppressor cells (MDSC), and cancer associated fibroblasts (CAF) in both high- and low-risk subgroups of MO-MLPS. ns. *p* > 0.05; * *p* < 0.05; ** *p* < 0.01; *** *p* < 0.001.

## Supplementary Tables

**Supplementary Table 1.** List of differential genes among novel subtypes of LUAD patients.

**Supplementary Table 2.** List of 100 genes specifically upregulated in each subtype.

**Supplementary Table 3.** TCGA and NCBI GEO datasets.

**Supplementary Table 4.** List of differentially expressed genes in each cell subset.

**Supplementary Table 5.** The Ro/e of each major cell type in LUAD samples.

**Supplementary Table 6.** The Ro/e of T, NK, B, and myeloid cell subsets in LUAD samples.

**Supplementary Table 7.** The C-index of the MO-MLPS in training and validation cohorts.

**Supplementary Table 8.** Features of MO-MLPS derived from the integrative construction of machine-learning algorithms in LUAD.

**Supplementary Table 9.** Compilation of 49 published signatures in LUAD obtained from the literatures.

**Supplementary Table 10.** C-index of MO-MLPS and 49 published prognostic signatures in LUAD.

**Supplementary Table 11.** Key resources table, including sequences of ANLN siRNAs and HA-dnANLN primers.
